# Characterization of the human skin resistome and identification of two microbiota cutotypes

**DOI:** 10.1186/s40168-020-00995-7

**Published:** 2021-02-17

**Authors:** Zhiming Li, Jingjing Xia, Liuyiqi Jiang, Yimei Tan, Yitai An, Xingyu Zhu, Jie Ruan, Zhihua Chen, Hefu Zhen, Yanyun Ma, Zhuye Jie, Liang Xiao, Huanming Yang, Jian Wang, Karsten Kristiansen, Xun Xu, Li Jin, Chao Nie, Jean Krutmann, Xiao Liu, Jiucun Wang

**Affiliations:** 1 BGI-Shenzhen, Shenzhen, China; 2 China National Genebank, Shenzhen, China; 3 State Key Laboratory of Genetic Engineering, Collaborative Innovation Center for Genetics and Development, School of Life Sciences, and Department of Dermatology, Huashan Hospital, Fudan University, Shanghai, China; 4 Human Phenome Institute, Fudan University, Shanghai, China; 5 IUF-Leibniz Research Institute for Environmental Medicine, Düsseldorf, Germany; 6 Department of Skin & Cosmetic Research, Shanghai Skin Disease Hospital, Shanghai, China; 7 Institute for Six-sector Economy, Fudan University, Shanghai, China; 8 Laboratory of Genomics and Molecular Biomedicine, Department of Biology, University of Copenhagen, Copenhagen, Denmark; 9 Guangdong Provincial Key Laboratory of Genome Read and Write, Shenzhen, China; 10 Research Unit of Dissecting the Population Genetics and Developing New Technologies for Treatment and Prevention of Dermatological Diseases (2019RU058), Chinese Academy of Medical Sciences, Shanghai, China; 11 Medical Faculty, Heinrich-Heine University, Düsseldorf, Germany; 12 Tsinghua Shenzhen International Graduate School, Tsinghua University, Shenzhen, China; 13 BGI Education Center, University of Chinese Academy of Sciences, Shenzhen, China; 14 Institute of Rheumatology, Immunology and Allergy, Fudan University, Shanghai, China

**Keywords:** Shotgun metagenomic sequencing, Skin microbiome, Gene catalog, Resistome, Antibiotic resistance genes (ARGs), *Moraxella osloensis*, Cutotypes

## Abstract

**Background:**

The human skin microbiota is considered to be essential for skin homeostasis and barrier function. Comprehensive analyses of its function would substantially benefit from a catalog of reference genes derived from metagenomic sequencing. The existing catalog for the human skin microbiome is based on samples from limited individuals from a single cohort on reference genomes, which limits the coverage of global skin microbiome diversity.

**Results:**

In the present study, we have used shotgun metagenomics to newly sequence 822 skin samples from Han Chinese, which were subsequently combined with 538 previously sequenced North American samples to construct an integrated Human Skin Microbial Gene Catalog (iHSMGC). The iHSMGC comprised 10,930,638 genes with the detection of 4,879,024 new genes. Characterization of the human skin resistome based on iHSMGC confirmed that skin commensals, such as *Staphylococcus spp*, are an important reservoir of antibiotic resistance genes (ARGs). Further analyses of skin microbial ARGs detected microbe-specific and skin site-specific ARG signatures. Of note, the abundance of ARGs was significantly higher in Chinese than Americans, while multidrug-resistant bacteria (“superbugs”) existed on the skin of both Americans and Chinese. A detailed analysis of microbial signatures identified *Moraxella osloensis* as a species specific for Chinese skin. Importantly, *Moraxella osloensis* proved to be a signature species for one of two robust patterns of microbial networks present on Chinese skin, with *Cutibacterium acnes* indicating the second one. Each of such “cutotypes” was associated with distinct patterns of data-driven marker genes, functional modules, and host skin properties. The two cutotypes markedly differed in functional modules related to their metabolic characteristics, indicating that host-dependent trophic chains might underlie their development.

**Conclusions:**

The development of the iHSMGC will facilitate further studies on the human skin microbiome. In the present study, it was used to further characterize the human skin resistome. It also allowed to discover the existence of two cutotypes on the human skin. The latter finding will contribute to a better understanding of the interpersonal complexity of the skin microbiome.

**Video abstract**

**Supplementary Information:**

The online version contains supplementary material available at 10.1186/s40168-020-00995-7.

## Background

The skin microbiota plays fundamental roles in maintaining skin homeostasis, and microbial dysbiosis is associated with the onset and progression of many common skin diseases [1–3]. A precise characterization of the microbiota with high resolution is essential to fully explore the potential of manipulating the microbiome to manage disease [4]. In this regard, profiling based on shotgun metagenomic sequencing has remarkable advantages when compared to phylogenic marker gene-based microbiota surveys. It allows for more precise recognition of skin microbiota across all kingdoms (bacteria, fungi, and viruses) with high resolution (species to strain level) and it can also provide first insight into their functional diversity. Based on metagenomic data sets, reference gene catalogs have been developed and found to be essential tools that greatly facilitate data analysis [5–7]. Accordingly, for the gut microbiome, a repeatedly updated and increasingly comprehensive gene catalog exists [8]. This is in contrast to the current microbial gene catalog for human skin. It mainly relies on the foundational work by the Human Microbiome Project (HMP) [9], which was based on samples collected from 12 healthy adults from North America. Given this limited population size and the recognition that skin microbial communities vary among ethnic groups [10, 11], it may be regarded as prototypic in nature.

In the present study, we, therefore, developed a more comprehensive, integrated catalog. To this end, we recruited 294 healthy individuals in Shanghai, China, and collected their skin microbiome from three anatomical sites in the face (forehead (Fh), cheek (Ck), and the back of the nose (Ns)). We analyzed the 822 samples of skin microbiome by metagenome shotgun sequencing, generating an average of 3.9 Gb paired-end reads (100 bp) for each of the skin sample on BGISEQ-500 platform, totaling 3.2 Tb of high-quality data that was free of human DNA contaminants (Table S1). These data were subsequently combined with the previously mentioned HMP data from North Americans [9, 12] in order to construct an inter-continental gene catalog. The resulting Integrated Skin Gene Catalogue allowed (i) the first large sample-based characterization of the human skin resistome and (ii) the discovery that on facial skin two defined patterns of the microbial network exist, for which we coined the term “cutotypes.” Each cutotype was associated with a distinct pattern of data-driven marker genes, functional modules, and clinical phenotypes.

## Results

### The construction of the integrated Human Skin Microbial Gene Catalog

**Fig. 1:**
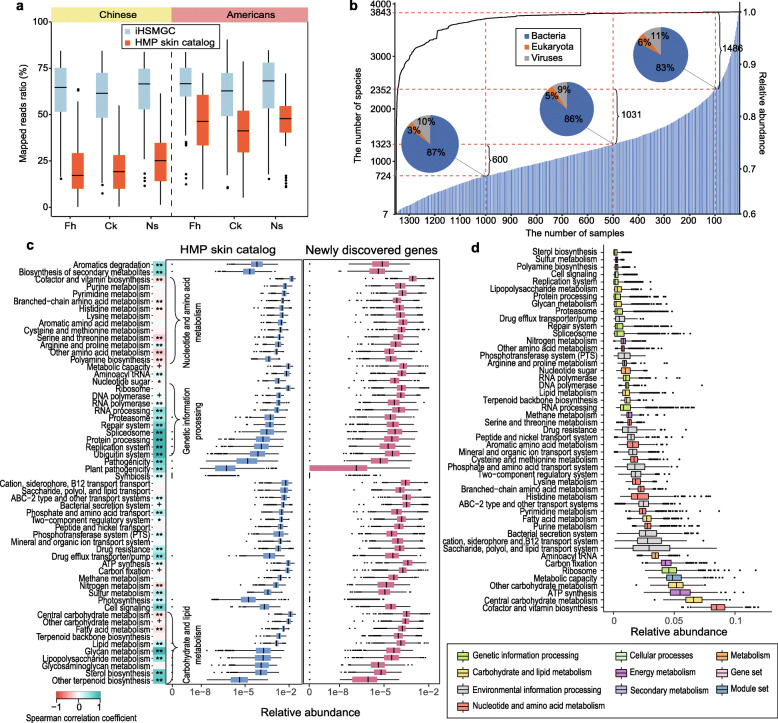
Evaluation of the integrated Human Skin Microbial Gene Catalog (iHSMGC). **a** Box plots comparing the reads mapping rate between the HMP skin catalog and the iHSMGC with two sets of sequencing data from this study (Chinese) and previous HMP study (Americans). Fh forehead, Ck cheek, and Ns the back of the nose. **b** The prevalence of certain microbial species in the population. The shadow bar chart (correspond to the left *Y*-axis) represents the number of microbial species that appeared across a certain number of samples (*X*-axis); the dashed lines separate the samples at sample size 1, 100, 500, 1000, respectively. The pie charts represent the corresponding proportion of bacteria, fungi, and viruses at the sample size 100, 500, 1000. The linear curve (correspond to the right *Y*-axis) quantifies the cumulative relative abundance of these species presented across the samples. **c** Box plot comparing the functional modules from the HMP gene catalog and newly identified genes in the iHSMGC. Box plots quantify the relative abundance of the genes within the corresponding functional module (vertical listed). The heat map next to the list represents the Spearman correlation coefficients between the genes in the HMP catalog and the genes newly identified within a certain functional module in terms of the gene abundance (+ *p* < 0.05, * *p* < 0.01, ** *p* < 0.001). **d** Box plot representing gene relative abundance from Han Chinese samples involved in a certain functional module. Different colors represent different functional modules in the KEGG KOs (B-level)

To construct an inter-continental gene catalog, we integrated our data with published raw data from HMP [9, 12], which led to a total of 1360 samples from 306 subjects, generating 4.3 Tb metagenomic sequencing data (Table S1). By using a newly established pipeline (Figure S1), we obtained the Human Skin Microbial Gene Catalog (iHSMGC) containing 10,930,638 genes. In comparison to the HMP gene catalog [9], 4,879,024 genes were newly identified in the iHSMGC. Each skin sample contained on average 501,756 genes, which is a bit less than the gene number reported for gut samples (762,665) [6]. More than 10% “trashed” reads from anatomical sites assessed in the HMP study [12] which correspond to Fh, Ch, and Ns could now be mapped and for these the average mapping rate was 60.01% (Fig. 1a and Table S1). In the HMP, samples were also obtained from other anatomical sites. When these data were mapped to iHSMGC, mapping rates were improved by 15.79% (for “moist” skin areas), 17.42% (for “sebaceous” skin areas), 30.78% (for “foot” skin areas), and 12.63% (for “dry” skin areas) (Figure S2a and Table S1). For Han Chinese samples, 40% more reads could be mapped to the iHSMGC than to the HMP catalog (Fig. 1a and Table S1), which might also reflect gene differences in the skin microbiota between Han Chinese and North Americans. When publicly available data from other shotgun metagenomic analysis of the human skin microbiome, including samples from patients with atopic dermatitis (AD) [13], psoriasis [14], and healthy children [15] were mapped to the iHSMGC, the average mapping rates were 62.45%, 72.26%, and 59.93%, respectively (Figure S2d). Richness estimation based on Chao2 [16] suggested that the iHMSGC covered most of the gene content in the sampled skin microbiome. This does not exclude; however, the possibility that the skin microbiome gene content will grow if more individuals and/or more skin sites will be sequenced (Figure S2c).

Next, we applied reference-based taxonomy annotation of the iHSMGC using the NCBI-NT database. 5,841,953 (53.45%) of the genes in the iHSMGC could be uniquely and reliably assigned to a phylum, 3,940,092 (36.05%) to a genus, and 3,219,956 (29.46%) to a species. Still, nearly half of the genes belonged to uncharacterized “microbial dark matter,” which may be derived from unknown taxa or genomic variations and may represent important gene content [9]. When assessing genome integrity (see “Methods” section), the iHSMGC covered on average, 82.13% of the microbial genomes for its top 10 abundant fungi genera, 78.96% of the virus genera, and 78.92% of the microbial genomes for the top 60 abundant bacteria genera (Figure S3b, c, d and Table S3). The average coverage of the most common fungi (*Malassezia*), bacteria (*Cutibacterium* and *Staphylococcus*), and viruses (*Propionibacterium virus* and *human papilloma virus*) in the skin was higher than 90% (Figure S3b, c, d and Table S3). At the strain level, common strains such as *Cutibacterium sp*., *Staphylococcus sp*., and *Malassezia sp.* reached a coverage of over 99.5% (Figure S3a, e and Table S3). Other skin bacteria such as *Streptococcus sp*., *Moraxella sp*., *Corynebacterium sp*., and *Ralstonia sp.* had coverages of more than 80% (Figure S3d, e, f and Table S3). Taken together, these results demonstrate that the iHSMGC is widely compatible and highly comprehensive.

Of note, by annotating phylogenetic composition according to the iHSMGC, we found that regardless of ethnic groups or anatomical sites, seven bacterial species were ubiquitously present across all samples. These included *Corynebacterium simulans*, *Cutibacterium acnes*, *Cutibacterium granulosum*, *Staphylococcus aureus*, *Staphylococcus capitis*, *Staphylococcus epidermidis*, *Streptococcus pneumonia*, and together they accounted for 60.8% of the microbial abundance (Fig. 1b). These species are likely to exert highly conserved functions in the human skin. In addition to these taxa, skin samples demonstrated high individual diversity of the microbial composition (Fig. 1b), similar to the phylogenetic profile in the human gut microbiota [17].

We next annotated the genes in the iHSMGC according to the Kyoto Encyclopedia of Genes and Genomes (KEGG). 10,964 KEGG orthologous groups (KOs) were identified from 6,415,308 genes (58.69% of the iHSMGC genes), which were assigned to 732 KEGG modules (Level D) under 49 main functional categories (Level C) (Fig. 1d). Among those newly identified 4,879,024 genes, 1,592,975 genes had functional annotation. Despite the enormous number of new genes, most of the new genes were still assigned to the previous categories. We observed that the functional potential related to microbial survival and growth showed no clear differences compared to the HMP dataset. A clear shift was detectable in some functional capacities, e.g., the nucleotides and amino acids metabolism and some carbohydrates metabolism (Fig. 1c). These differences may suggest functional diversity between the two ethnic groups.

### Antibiotic resistance genes in the skin microbiome

**Fig. 2:**
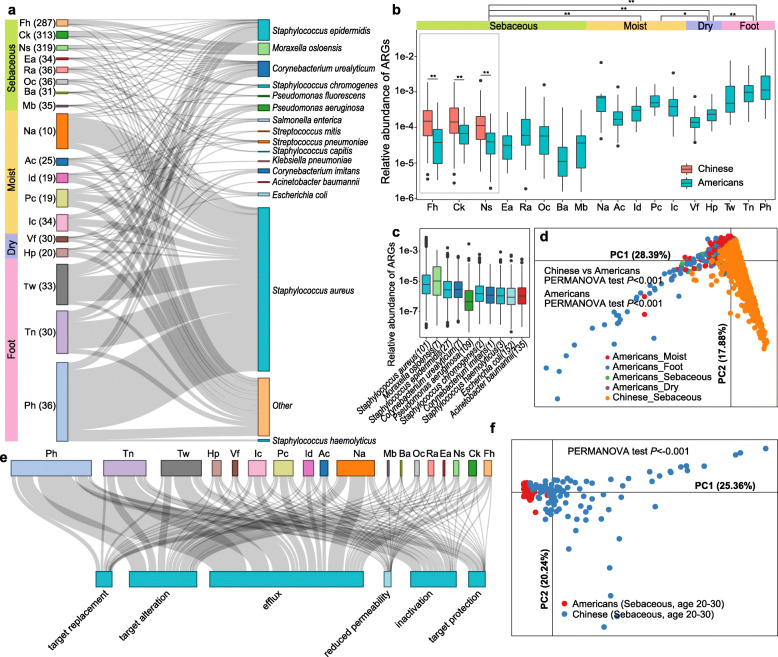
Antibiotic resistance genes (ARGs) in the skin microbiome. **a** Sankey diagram depicting the distribution of the top 10 antibiotic resistance bacteria ranked by the ARG abundance. The height of the rectangles indicates the relative abundance of the ARGs in the skin site (left) and species (right), different sites and species are indicated in different colors. Fh forehead, Ck cheek, Ns nose, Ea external auditory canal, Ra retroarticular crease, Oc occiput, Ba back, Mb manubrium, Na nare, Ac antecubital fossa, Id interdigital web, Pc popliteal fossa, Ic inguinal crease, Vf volar forearm, Hp hypothenar palm, Tw toe webspace, Tn toenail, Ph plantar heel. **b** Box plot comparing the relative abundance of ARGs in different body sites from Han Chinese and Americans. Venn diagram showing the ARG numbers in Chinese and Americans. **c** Box plot showing the relative ARG abundance of the top 10 antibiotic resistance bacteria ranked by ARG abundance. The exact number of ARGs in the species is noted in the brackets. **d** Principal component analysis demonstrating the patterns of ARG profile in different skin environments in Chinese and Americans. The PERMANOVA test was used to determine significance. **e** Sankey diagram depicting the distribution pattern of different resistance mechanisms deployed within a skin site. The rectangle’s area represents the relative abundance of ARGs in the sites or involved in the antibiotic resistance mechanisms. **f** Principal component analysis demonstrating the patterns of ARG profile in same skin sites and age ranges in Chinese and Americans. The PERMANOVA test was used to determine significance

The resistance of bacteria to antibiotic drugs is posing a major challenge to modern medicine. The collection of all the antibiotic resistance genes (ARGs) and their precursors which are expressed by both pathogenic and non-pathogenic bacteria has been termed the “resistome” [18]. The resistome has been intensively studied for gut-associated bacteria [18–21]. This is in contrast to the skin resistome, which has not yet been assessed in a large sample size. By capitalizing on the iHSMGC, we here provide the first large sample size-based characterization of the human skin resistome in Chinese and compare it with published North American data [9, 12]. We identified 3810 non-redundant ARGs to be distributed all over human skin (Table S4). Principal component analysis (PCA) based on resistome profiles showed significant separation among samples which were obtained from different skin environments (sebaceous, moist, dry, and foot) (Fig. 2d, PERMANOVA test, *p* < 0.05). The abundance of ARGs was highest in the foot areas and lowest in sebaceous regions (Fig. 2a, b) and thereby resembled the distribution of microbial diversity/species richness in these regions [12]. In the skin, the following six resistance mechanisms were found to be present with decreasing abundance: sequentially antibiotics efflux, followed by antibiotic target alteration, antibiotic inactivation, target protection, target replacement, and finally reduced permeability (Fig. 2e, Figure S4c).

Notably, the abundance of ARGs in Han Chinese was significantly higher than that in the North Americans (Fig. 2b). Moreover, the overall distribution of ARG genes in the two ethnic groups was significantly discrepant, including comparing samples of the same age and sampling sites between the two donor groups (Fig. 2d, f). 3418 non-redundant ARGs could be phylogenetically annotated to 456 microbial species. From these, we sorted the top 10 species by ARG abundance and found that ubiquitous skin commensals like *Staphylococcus aureus*, *Staphylococcus epidermidis*, and *Corynebacterium spp.* spread ARGs across all skin sites (Fig. 2a, c). Notably, two members of this top 10 list, i.e., *Acinetobacter baumannii* and *Pseudomonas aeruginosa*, were listed in the 2019 Antibiotic Resistance Threats Report (https://www.cdc.gov/drugresistance/index.html) and considered as multidrug-resistant “superbugs” that caused the majority of in-hospital mortality in the USA [22]. Our results show that such “superbugs” do exist in the skin of healthy Chinese and North Americans. Specifically, both species mainly presented in the foot region (Fig. 2a, c). Consistent with another study [23], we here confirmed that *Staphylococcus spp.* carried highly abundant ARGs (Fig. 2c), while another dominant commensal *Cutibacterium acnes* showed no ARGs.

The ARGs which we identified here to be present in skin are known to confer resistance to 38 classes of antibiotics (38 classes), of which fluoroquinolones (21.9%), tetracyclines (18.6%), and cephalosporins (7.8%) represent the most dominant ones (Figure S4a, b). Notably, these antibiotics are frequently used for skin-related indications, e.g., fluoroquinolones for the treatment of skin infections [24], tetracyclines for the management of acne and rosacea [25], and cephalosporins for the treatment of infected wounds and the prevention of skin infections after surgical procedures [26]. Consistent with the skin profiles of ARGs, PCA revealed significant separations between different antibiotics among different skin environments (sebaceous, moist, dry, and foot) (Figure S4d, PERMANOVA test, *p* < 0.05).

We next asked which factors beyond anatomical site might be associated with ARGs in human skins (Figure S5a). We found that the age of the individual from which the samples had been collected showed the strongest effect size (*R*^2^ ≈ 0.08, PERMANOVA test, *p* < 0.001) for ARGs among all variables (Figure S5a). Specifically, 25 classes of resistance potential were significantly correlated and mostly increased with age (Figure S5b). In addition, skincare habits also impacted on the abundance of ARGs (Figure S5a, c). A personal history of regularly applying skincare products was significantly associated with the increased abundance of ARGs against the free fatty acids, lincosamide, pleuromutilin, oxazolidinone, and streptogramin (Figure S5c).

### The composition of the facial skin microbiota in Han Chinese

**Fig. 3:**
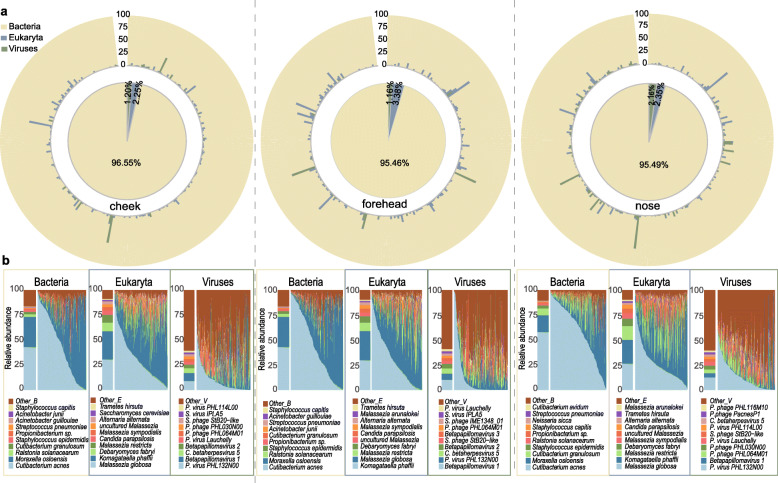
The composition of the facial skin microbiota in Han Chinese. **a** The relative abundance of microorganisms in the three kingdoms (bacteria, fungi, viruses) present in the three anatomical sites of the face. The central pie chart represents the average proportion of species in all samples and the outer circle depicts the proportions of species in each sample. **b** The compositions of the top 10 species of bacteria, fungi, and viruses in the three sites correspondingly. The bar chart on the left shows the average relative abundance of the species and the stacked area chart indicates the composition of species in each individual, ordered by the abundance of the top dominant species. Different colors represent different species

We next analyzed the microbial profile present in Han Chinese skin in a greater detail. In Chinese facial samples, bacteria, viruses, and fungi accounted for an average of 95.83%, 1.51%, and 2.66%, respectively (Fig. 3a). The most abundant fungal species were *Malassezia sp*., *Komagataella phaffii*, and *Candida parapsilosis*; for viruses *Propionibacterium phage*, *Betapillomavirus*, and *Staphylococcus phage* (Fig. 3b)*.* In general, the proportion of fungal and viral members present in Chinese samples was much lower than that reported for the same anatomical sites from the HMP dataset [9]. The most abundant bacteria in the Chinese samples were *Cutibacterium acnes*, *M. osloensis*, *Ralstonia solanacearum*, and *Staphylococcus epidermidis.* Of note, *M. osloensis* emerged as the second most abundant species in the Chinese samples. This is in marked contrast to North American samples, in which *M. osloensis* was detectable at only very low abundancy [27]. Considering the different sequencing platforms, we confirmed the high abundance of *M. osloensis* by analyzing the raw data from an independent shotgun sequencing dataset (Illumina HiSeq 2000) based on 40 samples from 40 Singapore Chinese (Figure S6) [13]. Taken together these results indicate a high abundancy of *M. osloensis* in Chinese, but not in North American skin, indicating microbial diversity between these two ethnic groups.

### Detection of microbiota-based cutotypes

**Fig. 4:**
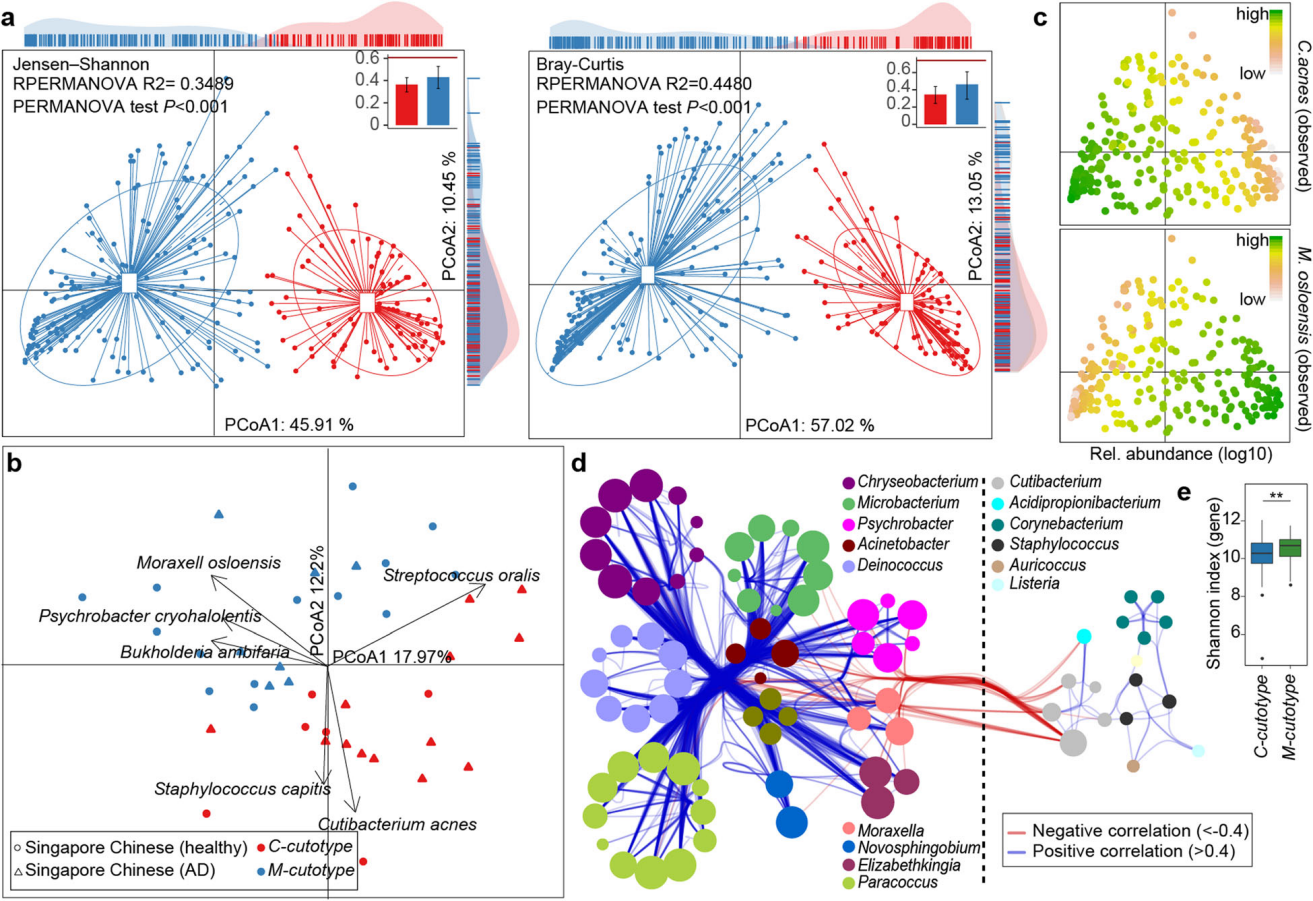
The skin microbial cutotypes and their phylogenetic differences. **a** PCoA using Jensen-Shannon distance and Bray-Cutis dissimilarity presenting the clustering of 247 samples from the forehead. Box plots in the top right show the mean distance within the corresponding groups in red or in blue. The red horizontal line indicates the average between-clusters distance. The PERMANOVA was calculated with adonis function in the vegan package to determine dissimilarity between two clusters as shown in the top panel. **b** PCoA analysis depicting the clustering of Singapore Chinese samples from a published dataset. Two principal components are plotted using the ade4 package in R with each sample represented by a filled circle or filled triangle. AD-atopic dermatitis. **c** Relative abundances of the two species *C. acnes* (upper) and *M. Osloensis* (lower). Based on PCoA results using Jensen-Shannon distances, the log_10_ of relative abundance in each sample was indicated by color. **d** Co-occurrence networks of the two cutotypes. Species enriched in the *M-cutotype* are shown on the left side, while species enriched in the *C-cutotype* are shown on the right side. Each node represents a species and the size of the node indicates the number of connections of the node to other nodes. Connect lines in red or blue indicate negative or positive correlation respectively. **e** Box plot showing the gene-based α-diversity (Shannon index) of the *M-cutotype* and the *C-cutotype* (** *p* < 0.01, Wilcoxon rank-sum test)

Similar to a previous study [9], the data provided here revealed enormous inter-individual microbial variations in the skin (Figure S7). We, therefore, asked if different individuals could be stratified according to their facial skin microbiota. To this end, we deployed multi-dimensional cluster analysis and principal coordinates analysis (PCoA). We discovered that the forehead skin samples from the 294 Han Chinese formed two distinct clusters (Fig. 4a and Table S5), for which we here coin the term “*cutotypes*.” We defined these two cutotypes by the dominance of one out of two species: *C. acnes* (referred to as “*C-cutotype*”) and *M. osloensis* (referred to as “*M-cutotype*”) (Fig. 4c). Differential analysis revealed that other microbes preferentially appeared within each cutotype. For example, *Moraxella bovoculi* and *Psychrobacter* sp. were enriched in the *M-cutotype*, while *Cutibacterium avidum*, *C. granulosum*, *Staphylococcus sp*., *Propionibacterium virus*, and *Staphylococcus phage* were enriched in the *C-cutotype* (Figure S8a). Species within one cutotype were highly correlated with each other in abundance (Fig. 4d), indicating stable ecological networks. Clustering into these two cutotypes was also applicable to facial skin sites other than the forehead, i.e., the back of the nose and the cheek (Figure S8e, f, Table S5). In fact, 69.64% of the tested individuals had identical cutotypes (either *M- or C-cutotype*) in all three facial sites (Table S5).

In order to test the robustness of this classification, we analyzed publicly available raw data from independent shotgun metagenomic studies, which had been conducted in East Asians by sampling non-facial skin sites. Accordingly, microbial samples from the right antecubital fossae [13] or from the neck/head region [15] also showed the existence of the *M-* as well as *C-cutotype* in East Asian skin (Fig. 4b and Figure S8b). In contrast, when skin microbiome samples from North Americans [9, 12] or Italians [14] were analyzed, these two cutotypes could not be well-detected. In such samples, we did observe, however, a tendency towards separation into different microbial patterns. These tendencies were driven either by *Propionibacterium sp.* or by a combination of *Staphylococcus sp.* with other species (Figure S8c, d).

### Function and clinical relevance of the cutotypes

**Fig. 5:**
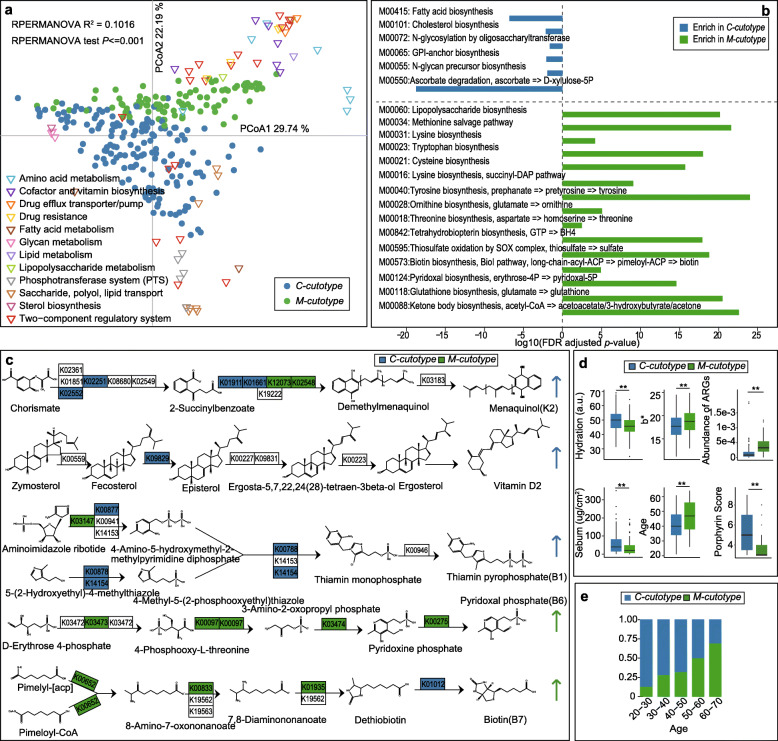
Functional module and clinical differences between the two cutotypes. **a** PCoA analysis depicting the distribution of differential KEGG-modules (Level-D) in terms of the relative abundance within the two cutotypes. Green and blue circles indicate the *M-cutotype* and *C-cutotype* samples, respectively. Triangles represent KEGG-modules, and different colors represent different KEGG-modules. **b** Bar chart demonstrating different enrichment of functional modules in the two cutotypes. **c** Reaction steps for the synthesis of five vitamins. KOs enriched in the *M-cutotype* are shown in green whereas the KOs enriched in the *C-cutotype* are shown in blue. The color of the arrow indicates the general enrichment of the KOs in the corresponding cutotype. **d** Box plots comparing the clinical parameters and the abundance of ARGs of the two cutotypes (** *p* < 0.01, Wilcoxon rank-sum test). **e** Bar chart presenting the composition ratio of the two cutotypes in different age groups

In order to better understand, the significance of these two cutotypes present in Chinese skin, we next assessed their functional module profiles. These studies revealed an enormous degree of functional disparity between the two cutotypes, which concerned functions related to metabolism and drug resistance (Fig. 5a). As an example, the two cutotypes were functionally diverse in vitamin biosynthesis: in the *C-cutotype* genes involved in the biosynthesis of menaquinone (vitamin K2), ascorbate (vitamin C), ergocalciferol (vitamin D2), and thiamine (vitamin B1) were enriched, whereas in the *M-cutotype* genes involved in the synthesis of pyridoxal (vitamin B6), biotin (vitamin B7 or H), cobalamin (vitamin B12), and riboflavin (vitamin B2) were more abundant (Fig. 5c and Table S6).

The two cutotypes also greatly differed for the enrichment of genes relevant to nutrition. In the *M-cutotype*, there was a substantial module enrichment in the metabolism of sulfur, aromatic compounds, and all kinds of amino acids (Fig. 5a, b, Figure S9 and Table S6). This was in sharp contrast to the *C-cutotype*, for which modules relevant for fatty acid biosynthesis and metabolism of carbohydrates and sterols were enriched. *C-cutotype* microbiota seemingly favored carbohydrates as their carbon source, because 17 types of the phosphotransferase system (PTS)-related functional modules, which are responsible for the translocation and phosphorylation of carbohydrate in prokaryotes [28] (Table S6) were enriched. This would be in contrast to *M. osloensis*, i.e., the dominant species in the *M-cutotype*, which previously has been described as a non-fastidious bacterium which was able to grow in a mineral medium supplemented with a single organic carbon source [29, 30]. Notably, *Moxarella sp.* was shown to be incapable of utilizing any carbohydrates or to possess any saccharolytic activity, but to strictly depend on other carbon sources such as acetic or lactic acid [29–32]. Our observations are thus consistent with the assumption that the two cutotypes have different “nutrient requirements.”

The two cutotypes also displayed distinct ARG patterns. Overall, the relative abundance of ARGs was markedly higher in the *M-* than the *C-cutotype* (Fig. 5d). Specifically, the *M-cutotype* exhibited a significant ARG enrichment conferring resistance to a broad spectrum of antibiotics (Figure S10a). In contrast, ARGs in the *C-cutotype* were enriched against only 3 classes: oxazolidinone, pleuromutilin, and lincosamide (Figure S10a). In general, the abundance of ARGs increased with age (Figure S5). After adjusting for age, the cutotype-related ARG abundance was still present (Figure S10b).

Finally, we asked if each of the two cutotypes would be associated with a distinct pattern of skin properties of the host. We found that *C-cutotype* skin was more hydrated and more oily. Accordingly, levels of skin surface sebum, as well as its microbial metabolite porphyrin [33], were increased. In contrast, *M-cutotype* skin was dryer, i.e., less hydrated, skin surface sebum levels were reduced, and the prevalence of the *M-cutotype* significantly increased with age (Fig. 5d, e and Table S7).

## Discussion

The iHSMGC is a comprehensive resource for further investigations of the skin microbiome, covering strains with a diverse range of population frequencies and abundance in the human skin. The construction of iHSMGC was similar to the method previously reported [6]. In order to improve the computational efficiency, iHSMGC was obtained through five-time clustering (Fig S1), which may overestimate the similarity among gene segments and discard non-redundant genes. It should also be noted that the average mapping rate of reads for samples (the USA and China) was 60.01%, and the average mapping rate was the same in other samples including diseases (psoriasis and dermatitis) and different age groups (children and adolescents). Therefore, we believe that iHSMGC is the most comprehensive gene catalog for skin microbiome to date.

In recent years, the role of the human microbiota as a reservoir of ARGs has received increasing attention. The vast majority of previous studies have focused on the gut [19–21] and a few on the lung microbiome [34]. Here, we report the first comprehensive large sample size analysis of the human skin resistome. The gut resistome mainly includes genes conferring resistance against tetracyclines, ß-lactams, aminoglycosides, and glycopeptides, followed by chloramphenicol and macrolides [34]. For the lung, the most abundant ARGs are ß-lactamases [34]. According to previously published data and the present study, ARGs in the skin mainly include fluoroquinolones, ß-lactamases, glycopeptides, aminoglycosides, macrolides, and tetracyclines resistance genes [9, 12, 23, 35].

We newly observed that the abundance of ARGS in Han Chinese was significantly higher than in North Americans. This difference likely reflects a more prevalent usage of antibiotics in the Chinese population, which might not be restricted to its use in clinics, but also in animal husbandry and fisheries [36, 37]. This assumption is supported by the present observation that certain ARGs such as Carbapenems-resistant genes were highly enriched in Chinese, but not in Americans. Accordingly, Carbapenems and other ß-lactam antibiotics are well known to be overused/misused in China [38]. Of note, we are aware that the two studies differ with regard to sampling and DNA purification protocols as well as the sequencing platforms (Table S9). Based on current literature [13, 39, 40], however, these technical and methodological differences are unlikely to account for the biological differences between Han Chinese and North Americans that we have observed in the present study.

In addition to ethnicity, the abundance of ARGs in skin was also significantly affected by age. This is similar to the age-dependent development of ARGs in the gut microbiome and likely reflects the fact that over a lifetime, exposure to antibiotics and thus the risk of developing resistance against antibiotics increases [41, 42]. We also newly observe that a history of regular application of skincare products also significantly influenced the abundance of ARGs. Many skincare products contain plant-derived extracts and exhibit antimicrobial activities [43], which may convey selection pressure for the enrichment of antibiotic-resistant strains and thus ARGs [20, 36]. This might also explain the present observation that the foot region showed the highest abundance and diversity for ARGs. It is exactly here where skincare products from other skin sites are thought to drip down along the body to concentrate and cause a high chemical diversity [44].

The skin resistome results of our study support the concept that the human skin microbiota constitutes a significant reservoir of ARGs accessible to pathogens [42]. The diversity of resistance genes in the human skin microbiome is likely to contribute to the future emergence of antibiotic resistance in human pathogens [34]. In this regard, the present discovery of superbugs being part of the human skin resistome in both Han Chinese and North American samples is of particular relevance.

The second most abundant species in Chinese samples was *M. osloensis*. This is in sharp contrast to North American samples, in which *M. osloensis* was detected only at very low abundancy. The reason for this ethnic difference might be the sample size. Surprisingly, *Enhydrobacter aerosaccus*, i.e., another species which has been repeatedly identified in Chinese skin via 16s rRNA microbial surveys [10, 45–47], was absent from our samples. By comparing the 16s rRNA sequence of the two species, we realized, however, that *M. osloensis* and *Enyhydrobacter aerosaccus* were 99.45% identical in the marker gene region. Considering the complete genome sequencing of *M. osloensis* isolated from the human skin was determined in 2018 [48], and former 16s rRNA sequence database [49] was absent from *M. osloensis* taxonomy, we, therefore, believe that it might have caused mis-annotations in previously published marker gene-based studies (Table S8). According to our data, *M. osloensis* represents a signature species of one of two cutotypes present in Chinese skin, with *C. acnes* indicating the other one. We found that each cutotype was associated with a distinct pattern of functional modules. Our results are consistent with known differences in the metabolism and nutritional requirements between the two dominant strains. Accordingly, *C. acnes* mainly use carbohydrates as their carbon source, which is reflected by the present observation that 17 functional modules (KEGG) in the phosphotransferase system (PTS) (Table S6), that is known to be responsible for carbohydrate translocation and phosphorylation in prokaryotes, were exclusively enriched in the *C-cutotype* microbiome. The phosphotransferase system is relevant for the capacity to metabolize glucose, maltose, lactose, fructose, and cellobiose and might thus reflect the dependence of the *C-cutotype* microbiota on the availability of carbohydrates [28]. In contrast, *M. osloensis*, the dominant species in the *M-cutotype*, was reported to be incapable of utilizing any carbohydrates, but strictly depend on other carbon sources such as acetic or lactic acid [29–32]. The two cutotypes also differed by functional annotation with regard to vitamin biosynthesis. Genes involved in menaquinone, ascorbate, ergocalciferol, and thiamine synthesis were more dominant in the *C-cutotype*, whereas genes involved in the synthesis of pyridoxal, biotin, cobalamin, and riboflavin appeared to be more relevant/abundant in the *M-cutotype* (Fig. 5c, Table S6). Taken together, these results indicate the existence of different microbial trophic chains in the skin, which might be responsible for the development of different communities of skin microorganisms and thus cutotypes.

In a previous study on the skin microbiota in patients with psoriasis the existence of two so-called “cutaneotypes” was reported, which were dominated either by *Proteobacteria* or *Actinobacteria* [50]. Given the fact that the microbial resolution of the cutaneotypes with 16s rRNA data was at Phyla level, and thus limited when compared to the species level with metagenomics data, which was used here to define the cutotypes, we would like to point out that the two terms have been defined differently and should not be used synonymously. Of note, the two cutotype-indicator species *Cutibacterium acnes* and *M.osloensis* belong to *Actinobacteria* and *Proteobacteria*, respectively, which have been used to define “cutanotypes.” Thus, the existence of cutaneotypes in psoriasis patients might be a cross-confirmation of the existence of distinct skin microbial communities within the human population, as indicated by the identification of two cutotypes in the present study.

Interestingly, the two cutotypes were also associated with distinct clinical phenotypes. In individuals with the *C-cutotype*, the facial skin showed a higher hydration status and increased sebum production (Fig. 5d). Also, microbial diversity was lower, which is consistent with the observation that sebaceous skin sites harbor less bacterial species (Fig. 5e) [9]. In contrast, the *M-cutotype* skin was less hydrated and less oily, but showed a higher species richness and biodiversity (Fig. 5d), thereby resembling older skin [51–54]. The *M-cutotype* was indeed positively associated with age, whereas the *C-cutotype* was more frequent in younger individuals (Fig. 5e, Table S7)**.** It should be noted, however, that both cutotypes could be identified in any age group, i.e., the *M-cutotype* was also detectable in young and middle-aged individuals, whereas the *C-cutotype* was also present in the elderly (Fig. 5e, Table S7).

The design of the present study does not allow to determine if the relationship between cutotypes and skin properties/phenotypes is mono- or bidirectional. Accordingly, a specific skin phenotype might not only define a cutotype, e.g., by providing the nutritional environment and thereby selection pressure for its development, but it might also—at least in part—result from the presence of a certain cutotype. The present observation that in *M-cutotype* skin, which phenotypically resembled aged skin, isocitrate lyase (*aceA*), and malate synthetase (*aceB*) genes were enriched, might indicate this possibility (Figure S11a). These genes are functionally relevant for the ability of *M. osloensis* to convert octylphenol polyethoxylates (OPEs) to alkylphenol ethoxylates (APEs) [48]. This constitutes an estradiol disrupting activity [55, 56], which might contribute to skin aging.

In addition to the hydration and sebum status of the skin, we also observed that individuals with the *M-cutotype* tended to have a more yellowish constitutive skin color. This phenotypical association might be due to the observed enrichment of functional modules relevant for beta-carotene biosynthesis (Figure S11b), which might reflect an increased production of ß-carotene by *M-cutotype*-associated species since increased ß-carotene levels are well known to cause a yellowish skin color [57].

Different from the previous host physiology-driven skin classification (sebaceous, moist or dry), we define “cutotype” as a microbiome-driven classification, which depicts the landscape characteristics of different microbial ecological homeostasis reached on the skin. Based on different types of microbe-networks and molecular signatures, we speculate that the selection pressure for the establishment of cutotypes is “nutrition,” which is reminiscent of the proposed model for the establishment of “enterotypes” [17, 58]. Whether the present cutotype-based stratification is of clinical significance is currently not known. It is, however, indicated by the present observation that ARGs are enriched in the *M-cutotype* skin. Also, the skin microbiota can affect xenobiotic metabolism, and this interaction might result in cutotype-dependent differences in skin drug metabolism [59] and thereby impact skin health.

## Conclusions

In this study, we have used shotgun metagenomic sequencing of a large number of samples to develop an iHSMGC. We believe that this catalog will prove to be a valuable tool for future studies to better understand the human skin microbiome. In the present study it allowed us (i) to comprehensively analyze the human skin resistome, (ii) to identify *M. osloensis* as a new dominant bacterium on the skin of Han Chinese, and (iii) to discover that based on skin microbial signatures, two cutotypes exist on the human skin.

We believed this classification of cutotypes would largely facilitate our understanding of microbial signatures from great interpersonal complexity without compromising the major influences from the microbiota, such as variant adaptation to topically applied drugs, cosmetics, and environmental noxae such as solar radiation and air pollution; therefore, it can be instructive to individualize measures towards the improvement of skin health into practice.

## Materials and methods

### Study population and microbial sampling

Forty-six male and 248 female healthy volunteers, who were 20 to 65 years old, were recruited from the general population in Shanghai between April and May 2017. Medical and medication history was obtained for each individual by questionnaires. Subjects with any history of skin diseases and intake of systemic or local antibiotics in the past 6 months were excluded. To maximize microbial skin load, each subject was instructed to wash the face only with tap water and to refrain from the application of any skin-care or cosmetic products on the sampling day before sampling.

Three skin sites (forehead, cheek, the back of the nose) were sampled for each subject. Study personnel wore sterile gloves for each sample collection. Samples were collected in a temperature and humidity-controlled room at 20 °C and 50% humidity. To obtain sufficient DNA from the three anatomical skin sites, which were low and variable in microbial biomass, and for the sake of establishing uniform standards between samples, a skin area of 4 cm^2^ was swabbed by sterile polyester fiber-headed swabs moistened with a solution of 0.15 M NaCl and 0.1% Tween 20 [60]. The sampling regions were swabbed 40 times each. Then, the swab head was fractured, placed in a sterilized 1.5 mL centrifuge tube, and stored at − 80 °C [9].

### Skin physiology assessment and skincare habit survey

Skin physiological parameters were collected in a temperature and humidity conditioned room (20 ± 1 °C, 50 ± 5% relative humidity) after an acclimatization period of 30 min for each study subject. The investigators for each device were fixed to avoid any personnel errors. Transepidermal water loss (TEWL) was measured employing a Vapometer® (Delfin Technologies Ltd, Kuopio, Finland). Skin hydration levels in the stratum corneum were determined with a MositurMeter D Compact device (Delfin Technologies Ltd, Kuopio, Finland). Sebum was determined by Sebumeter® SM815 (Courage & Khazaka electronic GmbH, Cologne, Germany). The level of sebum was expressed as μg/*cm*^2^. Skin pH was measured with Skin-pH-Meter PH 900 (Courage & Khazaka electronic GmbH, Cologne, Germany). Skin color (L*a*b) and pore were assessed by ImageJ software based on photos obtained from the VISIA-CR (Canfield Scientific Inc, Fairfield, NJ). The value increase for L*(lightness) represents from black to white; the value for a* is from green to red; the values for b* indicate blue to yellow. Porphyrin was visually graded according to the reference image on a scale from “1” to “3” based on the VISIA-CR photos. In this scale, “1” to “3” represent mild/moderate/severe deposition of porphyrin under the UV light source. The final score of the porphyrin, on a 3–9 scale, is the sum of scores from three trained persons based on the above scoring criteria. The frequency of skincare was obtained from volunteers by questionnaire; here, we mainly considered the frequency, whereas the detailed skincare products were not taken into consideration.

### DNA extraction and metagenomic sequencing

#### DNA extraction and whole genome amplification

DNA was extracted following the MetaHIT protocol, as previously described [40]. The extracted DNA from all samples was amplified to reach the requirement for subsequent library construction by PicoPLEX WGA Kit (Rubicon) following the manufacturer’s protocol. The DNA concentration was quantified by Qubit (Invitrogen).

#### Library preparation and sequencing

A 500 ng of input DNA was fragmented ultrasonically with Covaris E220 (Covaris, Brighton, UK), yielding 300 to 700 bp of fragments. Sheared DNA without size selection was purified with an Axygen^TM^ AxyPrep^TM^ Mag PCR Clean-Up Kit. An equal volume of beads was added to each sample, and DNA was eluted with 45 *μ*L TE buffer. Twenty nanograms of purified DNA was used for end-repairing and A-tailing with a 2:2:1 mixture of T4 DNA polymerase (ENZYMATICS^TM^ P708–1500), T4 polynucleotide kinase (ENZYMATICS^TM^ Y904–1500), and Taq DNA polymerase (TAKARA^TM^ R500Z) which was heat-inactivated at 75 °C. Adaptors with specific barcodes (Ad153 2B) were ligated to the DNA fragments by T4 DNA ligase (ENZYMATICS^TM^ L603-HC-1500) at 23 °C. After the ligation, PCR amplification was carried out. Fifty-five nanograms of purified PCR products was denatured at 95 °C and ligated by T4 DNA ligase (ENZYMATICS^TM^ L603-HC-1500) at 37 °C to generate a single-strand circular DNA library. Sequencing was performed according to the BGISEQ-500 protocol (SOP AO) employing the paired-end whole-metagenome sequencing (WMS) mode, as described previously [61].

### Public data used

In addition to our sequencing data, we downloaded skin metagenomic data from HMP [12] (SRA under bio-project 46333) to construct the iHSMGC. The public data from HMP comprised 539 skin metagenomic samples from 18 body sites of 12 healthy volunteers: Alar crease (AI), Cheek (Ck), Forehead (Fh), External auditory canal (Ea), Retroauricular crease (Ra), Occiput (Oc), Back (Ba), Manubrium (Mb), Nare (Na), Antecubital fossa (Ac), Interdigital web (Id), Popliteal fossa (Pc), Inguinal crease (Ic), Tow webspace (Tw), Plantar heel (Ph), Toenail (Tn), Plantar heel (Ph), Volar forearm (Vf), and Hypothenar palm (Hp). The body sites were grouped into four types: sebaceous (AI, Ck, Fh, Ea, Ra, Oc, Ba, and Mb), moist (Na, Ac, Id, Pc, and Ic), foot (Tn, Tw, and Ph), and dry (Vf and Hp). To validate the general significance of iHSMGC and cutotypes, we also downloaded metagenomic data from studies in allergic dermatitis (AD) [13], psoriasis [14], and children [15] from NCBI with the accession no. PRJNA277905, no. PRJNA281366, and no. PRJEB26427, respectively.

### Gene catalog construction and gene annotation

#### Gene catalog construction

To construct the skin microbiome gene catalog, sequencing reads from this study as well as from HMP were processed (quality control, removal of human sequences, assembling, gene prediction) using the pipeline shown in Supplementary Fig. 1. SOAPnuke [62] was used for quality control. SOAPaligner2 [63] was for identifying and removing human sequences if they shared > 95% similarity with the human genome reference sequence (hg19) [11]. Consistent with previous findings, on average 80% reads were from human origin instead of microorganisms (Supplementary Fig. 2b). High-quality reads were used for de novo assembly via SPAdes (version 3.13.0) [64], which generated the initial assembly results based on different k-mer sizes (*k* = 21, 33, 55, 77,99). Ab initio gene identification was performed for all assembled scaffolds by MetaGeneMark (version 3.26) [65]. These predicted genes were then clustered at the nucleotide level by CD-HIT (version 4.5.4), CD-HIT parameters are as follows: - G 0 - M 90000 - R 0 - t 0 - C 0.95 - as 0.90 [66], genes sharing greater than 90% overlap and greater than 95% identity were treated as redundancies. Thus, we obtained a two cohorts non-redundant gene catalog (2CGC) including 13,324,649 genes. To further ensure the integrity of the gene catalog, we did the following: first, sequence alignment was carried out between 2CGC and National Center for Biotechnology Information non-redundant nucleotide (NCBI-NT, downloaded at Aug. 2018): 931 genera genomes (including 2,761 prokaryotes, 112 fungi, 479 viruses)—were identified to be existing in 2CGC (Table S2); we then downloaded the genomes or draft genomes of these microbes and used MetaGeneMark to predict the coding regions; these predicted genes were later pooled, and the software CD-HIT was used to remove the redundant genes. Thus, we got 7,496,818 non-redundant genes, which we refer to as the sequenced gene catalog (SGC). Finally, the gene catalogs based on 2CGC and SGC were combined using CD-HIT. Genes existing in at least ten samples were selected to form the final iHSMGC, which comprised 10,930,638 genes.

#### Assessment of iHSMGC genome integrity

To evaluate the genome integrity of a single microbe in iHSMGC, we constructed draft microbial reference genomes of 5409 bacteria, 2023 viruses, and 158 fungi (https://ftp.ncbi.nlm.nih.gov/genomes/) and sequenced alignment iHSMGC with the database. The definite means were as follows: (1) predicting the coding sequence (CDS) of genomes and (2) map iHSMGC with genome CDS using the BWA MEM method (default parameter). The coverage of each genomic CDS region was obtained.

#### Taxonomic classification of genes

Taxonomic classification of genes was performed based on the National Center for Biotechnology Information non-redundant nucleotide (NCBI-NT, downloaded at Aug. 2018) database. We aligned about 11 million genes of iHSMGC onto the NCBI-NT using BLASTN (v2.7.1, default parameters except that -evalue 1e-10 outfmt 6 -word_size 16). At least 70% alignment coverage of each gene was required. For multiple best-hits (from NCBI-NT database) mapping for the same gene with the same %identity, *e* value and bit score, we have used the following strategy:

We performed statistics on multiple best-hits (from NCBI-NT database) mapping for the same gene, including the number of annotated species present, the number of occurrences of each annotated species, and the average similarity of the same species. After completion of the statistics, the species annotation with the highest frequency and the highest average similarity was defined as the annotation of the gene. In case that different species for a single gene ranked the same in the statistics, we have chosen the species annotation that ordered first (i.e., the order of blast hits and *e* value). Accordingly, 95% identity was used as the critical value for species assignment, 85% identity was used as the critical value for genus assignment, and 65% for phylum assignment [6]. The 3.97 million genes of the gene catalog were annotated taxonomically.

#### Functional annotation of genes

We aligned putative amino acid sequences, which translated from the iHSMGC, against the proteins or domains in KEGG databases (release 84.0, genes from animals or plants were excluded) using BLASTP (v2.7.1, default parameters except that -outfmt 6 -evalue 1e-6). At least 30% alignment coverage of each gene was required. Each protein was assigned to a KEGG orthologue (KO) based on the best-hit gene in the database. Using this approach, 6.42 million of the genes in the combined gene catalog could be assigned a KO.

#### Quantification of genes

The high-quality reads from each sample were aligned against the gene catalog by SOAP2.21 with the criterion of identity > 90% [63]. In our sequence-based profiling analysis, the alignments that met one of the following criteria as previously described could be accepted [67]: (i) an entire of a paired-end read can be mapped onto a gene with the correct insert-size and (ii) only when the one end of paired-read was mapped outside the genic region; the other end of reads can be mapped onto the end of a gene. In both cases, the mapped read was counted as one copy. The formula used in this study for calculating gene relative abundance is similar to RPKM/FPKM (reads per kilobase of exon model per million mapped reads/fragments per kilobase of exon model per million mapped fragments) value. Accordingly, for any sample 푆, we calculated the abundance as follows:

Step 1: Calculation of the copy number of each gene:

$$ {b}_i=\frac{x_i}{L_i} $$

Step 2: Calculation of the relative abundance of gene i:

$$ {a}_i=\frac{b_i}{\sum_j{b}_i}=\frac{\frac{x_i}{L_i}}{\sum_j\frac{x_i}{L_i}} $$

*a*_*i*_: The relative abundance of gene i in sample S

*L*_*i*_: The length of gene i

*x*_*i*_: The times which gene i can be detected in sample S (the number of mapped reads)

*b*_*i*_: The copy number of gene i in the sequenced data from S.

*j*: The iHSMGC gene number.

The value of *b*_*i*_ standardizes the effect of gene length in Step 1. The value of $\frac{b_i}{\sum_j{b}_i}$ standardizes the effect of sequencing depth in Step 2.

#### Construction of phyla, genera, species, and KO profiles

The relative abundances of phyla, genera, species, and KOs were calculated from the relative abundance of their respective genes using previously published methods [68]. For the species profile, we used the phylogenetic assignment of each gene from the original gene catalog and summed the relative abundance of genes from the same species to generate the abundance of that species. The phyla, genera, and KO profile were constructed using the same methods.

#### Rarefaction curve analysis

We used a rarefaction curve to assess the gene richness in our cohorts. For each given number of samples, we performed random sampling 100 times in the cohort with replacement. Moreover, we estimated the total number of genes that could be identified from these samples with the Chao2 index [69].

#### Determination and annotation of antibiotic resistance genes

Antibiotic resistance genes (ARGs) were identified using the Resistance Gene Identifier (RGI, v4.2.2) with default parameters and the CARD database (The Comprehensive Antibiotic Resistance Database, v3.0.7) [70]. DIAMOND was utilized for alignment [71]. In order to identify the species origins of drug resistance genes, the similarity of the predicted ARG segments to known species was estimated by aligning the predicted ARGs to the NCBI-NT using BLASTN (v2.7.1, default parameters except that -evalue 1e-10 outfmt 6 -word_size 16), and identified genes had an alignment coverage greater than 70%.

### Comparison of *Moraxella osloensis* and *Enhydrobacter aerosaccus*

To assess if the previously reported *Enhydrobacter aerosaccus* is, in fact, *Moraxella osloensis*, we used the following methods: (1) We downloaded 16S sequences of *Moraxella osloensis* (NR_104936.1) and *Enhydrobacter aerosaccus* (MH715214.1) from NCBI, the two sequences were aligned by BLASTN (v2.7.1, default parameters except that -evalue 1e-10 outfmt 6 -word_size 16), and found that the similarity between them can reach 99.450%. (2) We aligned the sequences annotated as *Enhydrobacter* in Greengene [49] with NCBI-NT using BLASTN and found that 78.9% of the sequences were annotated as *Moraxella osloensis*. (3) Using the same method, we found that 99.4% of the sequences annotated as *Enhydrobacter* in the MetaPhlAn2 [72] database were annotated as *Moraxella osloensis*.

### Statistical analysis

#### Multivariate analysis

Multivariate statistical analyses (PCA, PCOA) were applied to assess the skin microbiome within individuals. Principle component analysis (PCA) was performed on the three facial sites as previously described, using the ade4 package [73] in the R platform. Principle coordination analysis (PCOA) was performed based on the Jensen-Shannon distance (JSD)/Bray Curtis distance on the skin microbial composition and functional profile using the ade4 package [73].

#### Hypothesis test and multiple test correction

Wilcoxon rank-sum tests were performed to detect differences in the skin physiological and microbial characteristics between the three facial sites, including clinical parameters, gene count, Shannon index, and the relative abundances of species, KOs, and modules. For a certain phenotype feature (male/female), Fisher’s exact test was used. Unless otherwise indicated, *P* values were adjusted using the FDR correction by fdrtool package [74] in R. Statistical significance was set as adjusted *P* value < 0.05. Differentially enriched KEGG modules and KOs were identified, according to FDR adjusted *P* values. We used Wilcoxon rank-sum tests to obtain *P* values. FDR adjusted *P* values of less than 0.05 was used as the detection threshold for significance.

#### Permutational multivariate analysis of variance

The permutational multivariate analysis of variance (PERMANOVA) [75] was used to assess the effect of different covariates, such as cutotypes, age, sex, physicochemical index, and skin image information on all types of profiles. We performed the analysis using the method implemented in R package (vegan) [76], and 1000 times permutations to obtain the permuted *P* value.

### Biodiversity and richness analysis: α-diversity

The α-diversity (within-sample diversity) was calculated to estimate the gene diversities of each sample using the Shannon index [77]:

$$ {\mathrm{H}}^{\prime }=-\sum \limits_{i=1}^S{a}_i{lna}_i $$

where S is the number of genes and a_i_ is the relative abundance of gene i. A high α-diversity indicates a high evenness or many types of genes present in the sample.

### Cutotype: clustering and classification

To define a cutotype based on the skin microbiome, samples from each facial site were clustered using Jensen-Shannon distance (JSD) [78], respectively, which was calculated by taking the square root of the Jensen-Shannon divergence. The Jensen-Shannon divergence was an effective measure of divergence between distribution accounting for both the presence and abundances of microbes. Moreover, JSD was calculated according to this formula:

$$ \mathrm{D}\left(\mathrm{a},\mathrm{b}\right)=\sqrt{JSD\left({p}_a,{p}_b\right)}, $$

where

$$ \mathrm{JSD}\left(\mathrm{x},\mathrm{y}\right)=\frac{1}{2} KLD\left(x,\frac{x+y}{2}\right)+\frac{1}{2} KLD\left(y,\frac{x+y}{2}\right) $$

$$ \mathrm{KLD}\left(\mathrm{x},\mathrm{y}\right)=\sum \limits_i{x}_i\mathit{\log}\frac{x_i}{y_i} $$

In this formula, pa and pb are the abundance distributions of samples a and b, and KLD is the Kullback-Leibler divergence.

As described in the enterotyping tutorial (http://enterotype.embl.de/enterotypes.html), clustering was performed via partitioning around medoid (PAM) by the *pam* function in cluster package [79] in R. The optimal number of clusters was determined by the Calinski-Harabasz (CH) index:

$$ {CH}_k=\frac{B_k/\left(k-1\right)}{W_k/\left(n-k\right)}, $$

where *k* is the number of clusters, *n* is the number of data points, *B*_*k*_ is the between-cluster sum of squares (i.e., the squared distances between all points *i* and *j*, for which *i* and *j* are not in the same cluster) and *W*_*k*_ is the within-cluster sum of squares (i.e., the squared distances between all points *i* and *j*, for which *i* and *j* are in the same cluster). The CH index was calculated using clusterSim package [80] in R. Principal coordinates analysis (PCoA) was used to show cutotype results by the *cmdscale* function in R. The cutotype results were also verified based on Bray-Curtis (BC) distance using vegan package [76] in R. The JSD and BC of intra- and inter-cluster were shown by boxplots. We used the same method to define cutotype based on public data mentioned before for confirming the extensive existence of cutotype.

## Supplementary Information

**Additional file 1: Table S1.** Statistics for sequencing data of the 822 samples from Shanghai-China and the 538 HMP samples. **Table S2.** Description of the genus-level bacteria associated with the skin. **Table S3.** Detailed information on the genome coverage obtained by iHSMGC. **Table S4.** Detailed result of ARGs identified in iHSMGC. **Table S5.** Detailed information about the cutotype classification. **Table S6.** Microbial functions of significant difference between C-cutotype and M-cutotype. **Table S7.** Detailed distribution of cutotypes in different age groups. **Table S8.**. Detailed information on the comparison of *Moraxella osloensis* and *Enhydrobacter.*

**Additional file 2: Figure S1.** Construction of the iHSMGC (integrated Human Skin Microbial Gene Catalog). The metagenomic sequencing data from the Chinese and North American cohorts were processed with an in-house pipeline to generate their respective gene catalogs. The two catalogs were merged to form a Two Cohorts nonredundant Gene Catalog (2CGC). Sequenced microbial genomes or draft genomes coverage by 2CGC were regarded as potentially containing sequences of human skin origin. Therefore, microbial genomes were filtered by 2CGC, and the retained microbial genomes were then used to generate the SGC. Finally, the 2CGC was merged with the skin gene catalog (SGC) to generate the iHSMGC.

**Additional file 3: Figure S2.** Host information, coverage and completeness of the iHSMGC. a, Box plot comparing the reads mapping rate of the HMP dataset between HMP skin catalog and the iHSMGC. b, The Bee swarm plot showing the percentage of sequenced reads mapping to human hg19 of each sample. Different anatomical sites are indicated by different colors. c, Rarefaction curve based on gene profiles of 1,361 samples using the Chao2 estimator. d, Box plots demonstrating the read mapping rate from the dataset of Singapore Chinese (NCBI No. PRJNA277905), Italians (NCBI No. PRJNA281366) and another Singapore Chinese (NCBI No. PRJEB26427) by using the iHSMGC. AD-atopic dermatitis. Ea-External auditory canal, Ra-Retroarticular crease, Oc-Occiput, Ba-Back, Mb-Manubrium, Na-Nare, Ac-Antecubital fossa, Id-Interdigital web, Pc-Popliteal fossa, Ic-Inguinal crease, Vf-Volar forearm, Hp-Hypothenar palm, Tw-Toe webspace, Tn-Toenail, Ph-Plantar heel.

**Additional file 4: Figure S3.** Evaluation of iHSMGC integrity. Genome coverage of (a) *Malassezia sp.*, (b) top 10 genera of fungi, (c) top 25 genera of viruses, (d) top 60 genera of prokaryotes, each dot in (b-d) represents a species in the genera. Genome coverage of (e) *Cutibacterium sp*. and *Staphylococcus sp.,* (f) *Moraxella sp.* and (g) *Streptococcus sp.*

**Additional file 5: Figure S4.** Drug-resistant spectrum based on ARGs in different skin sites. a, Sankey diagram depicting the distribution of the top 15 types of antibiotics ranked by the corresponding ARG abundance. The height of the rectangles indicates the ARGs relative abundance against the corresponding drug resistance potential within the site. Each site and drug resistance potential is indicated in distinct colors. Fh-Forehead, Ck-Cheek, Ns-Nose, Ea-External auditory canal, Ra-Retroarticular crease, Oc-Occiput, Ba-Back, Mb-Manubrium, Na-Nare, Ac-Antecubital fossa, Id-Interdigital web, Pc-Popliteal fossa, Ic-Inguinal crease, Vf-Volar forearm, Hp-Hypothenar palm, Tw-Toe webspace, Tn-Toenail, Ph-Plantar heel. b-c, The pie chart showing the proportion of drug resistance (b) and resistance mechanisms (c). d, Principal component analysis indicating separation of drug-resistant spectrum within the different anatomical sites.

**Additional file 6: Figure S5.** Factors correlated with drug resistance potential in Chinese. a, Bar chart comparing the explained variance (R2) of factors impacting the relative abundance of ARGs using the Adonis test. The L-value represents skin color from dark to white, the b-value is skin color from blue to yellow. b, Bar chart depicting the types of antibiotics corrected with age by the Spearman correlation. c, Bar chart showing the correlation with skincare habit (*p* < 0.05).

**Additional file 7: Figure S6.** Differences in skin microbiota between Singapore Chinese and North Americans. a, Principal component analysis presenting the separation of skin microbiota of Singaporean Chinese (NCBI No. PRJNA277905) versus North Americans (HMP SRA bio-project 46333). The microbes, which were the main contributors to the separation, are indicated by arrows. b, The boxplot showing the prominent species that differ significantly in abundance between Singaporean Chinese and North Americans.

**Additional file 8: Figure S7.** Intraindividual differences are smaller than interindividual differences. Boxplots of Bray-Curtis distance depicts the similarity between anatomical sites in the face of the same individuals (intraindividual comparisons) or between the same/different sites of different individuals (interindividual comparisons). The left side of the dotted line shows the intraindividual differences, the right side the interindividual differences. The significance levels in the Wilcoxon rank-sum test are: +, *p* < 0.05; *, *p* < 0.01; **, *p* < 0.001.

**Additional file 9: Figure S8.** Microbial composition of the cutotypes and further validation. a, Heat map depicting the species differentially abundant within the two cutotypes (Wilcoxon rank-sum test, *p* < 0.01). Each lattice represents the relative abundance of the microbe in a sample, yellow indicates high abundance and blue indicates lower abundance. b-d, PCoA using Jensen-Shannon distance presenting the clustering of (b) samples from the Singaporean dataset (NCBI No. PRJEB26427), (c) Samples from the Italian (NCBI No. PRJNA281366) and (d) Samples from the HMP (SRA bio-project 46333). e-f, PCoA using Jensen-Shannon distance and Bray-Cutis dissimilarity presenting the clustering of samples from the cheek (e) and the back of the nose (f) of Han Chinese. Box plots in the top right show the mean distance within the corresponding groups in red or in blue. The red horizontal line indicates the average between-clusters distance. The PERMANOVA test was used to determine the significance between two clusters and is shown in the top left.

**Additional file 10: Figure S9.** Microbial functional differences between the two cutotypes. Using log10 (FDR adjusted *p*-value) bar-plot comparing the abundance of module (amino acid metabolism, lipopolysaccharide metabolism, cofactor biosynthesis, sulfur metabolism, glycan metabolism, sterol biosynthesis, and fatty acid metabolism) KOs of the two cutotypes present in the forehead areas. Green color indicates KO enrichment in the *M-cutotype* and blue means the enrichment in the *C-cutotype*. The color shade indicates the level of significance, i.e. dark green or dark blue equal the FDR adjusted *p*-value < 0.05, which is the threshold for a significant difference.

**Additional file 11: Figure S10.** Characteristics of different skin microbial cutotypes a, Alterations in skin microbial ARGs antibiotics and ARGs mechanism. b, The boxplot showing the differences in the abundance of ARGs between different age groups. Blue, *C-cutotype*-enriched; green, *M-cutotype*-enriched. The significance levels in the Wilcoxon test are denoted as: **, *p* < 0.01.

**Additional file 12: Figure S11.** Octylphenol polyethoxylates transformation and beta-carotene biosynthesis between the two skin cutotypes. a, Reaction step for the conversion of octylphenol polyethoxylates to alkylphenol ethoxylates. The histogram compares the abundance of the genes encode for the two enzymes within the two cutotypes (* *p*< 0.05, ** *p* < 0.01; Wilcoxon test). b, Reaction steps for the biosynthesis of beta-carotene in microorganisms. The green box represents the enrichment of the KOs in the *M-cutotyp*e, the white box represents no significant difference.

## Data Availability

The sequencing data from this study have been deposited in the CNSA (https://db.cngb.org/cnsa/) of CNGBdb with accession number CNP0000635 and NODE (https://www.biosino.org/node/index) with accession number OEP001168. A website (https://db.cngb.org/microbiome/genecatalog/genecatalog/?gene_name=Human%20Skin%20(10.9M)) has been set up to better visualize the annotation information of the gene catalog and guide researchers who are interested in using our data set and downloading specific sets of data.
