# Supplementary figures and images for: Characterization of the human skin resistome and identification of two microbiota cutotypes
Source: Microbiome. 2021 Feb 17;9:47. doi: 10.1186/s40168-020-00995-7 (PMC7890624; doi:10.1186/s40168-020-00995-7)

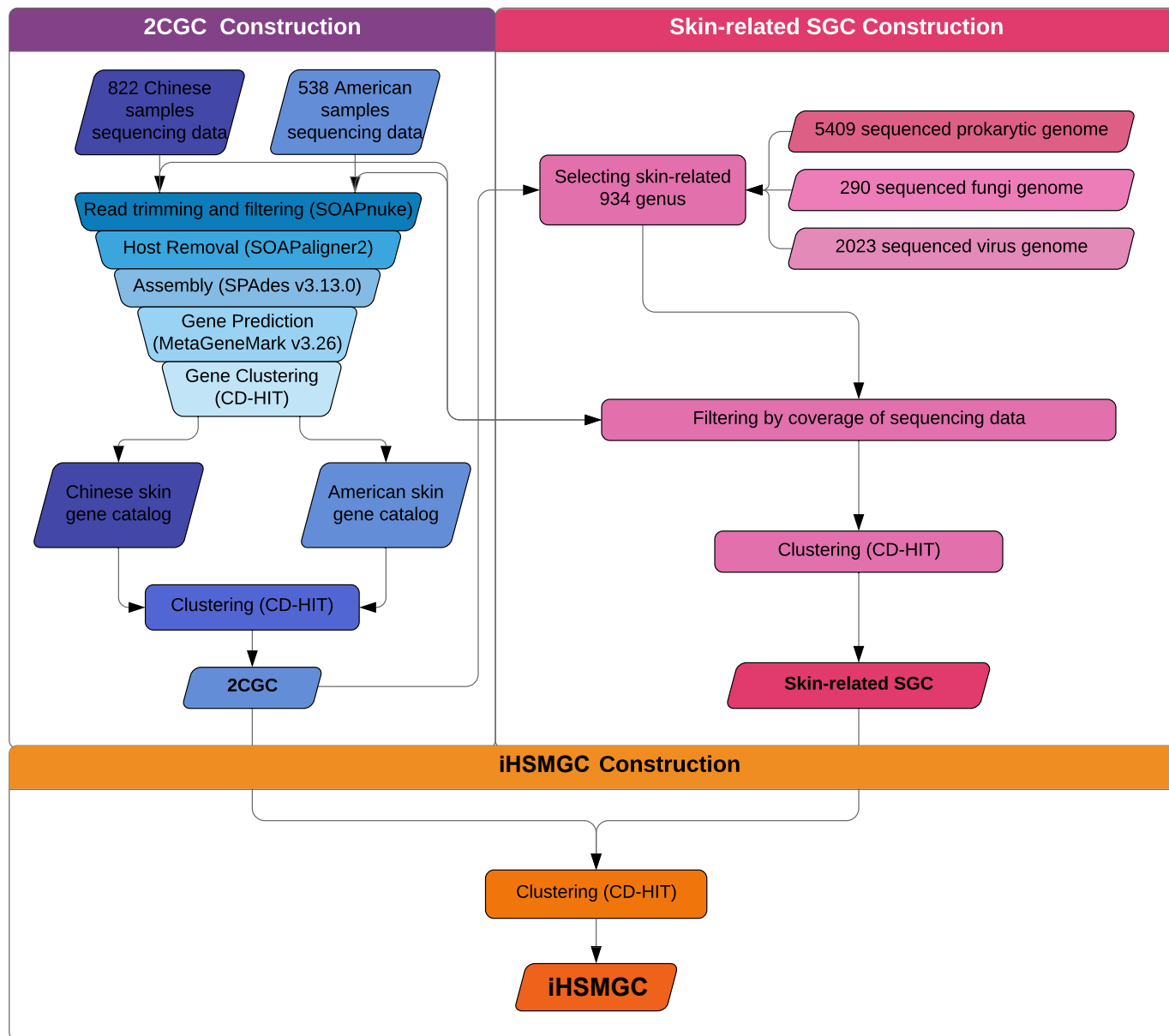

Supplement: Supplementary file 3 — Additional file 2: Figure S1. Construction of the iHSMGC (integrated Human Skin Microbial Gene Catalog). The metagenomic sequencing data from the Chinese and North American cohorts were processed with an in-house pipeline to generate their respective gene catalogs. The two catalogs were merged to form a Two Cohorts nonredundant Gene Catalog (2CGC). Sequenced microbial genomes or draft genomes coverage by 2CGC were regarded as potentially containing sequences of human skin origin. Therefore, microbial genomes were filtered by 2CGC, and the retained microbial genomes were then used to generate the SGC. Finally, the 2CGC was merged with the skin gene catalog (SGC) to generate the iHSMGC. [file 40168_2020_995_MOESM3_ESM.pdf]

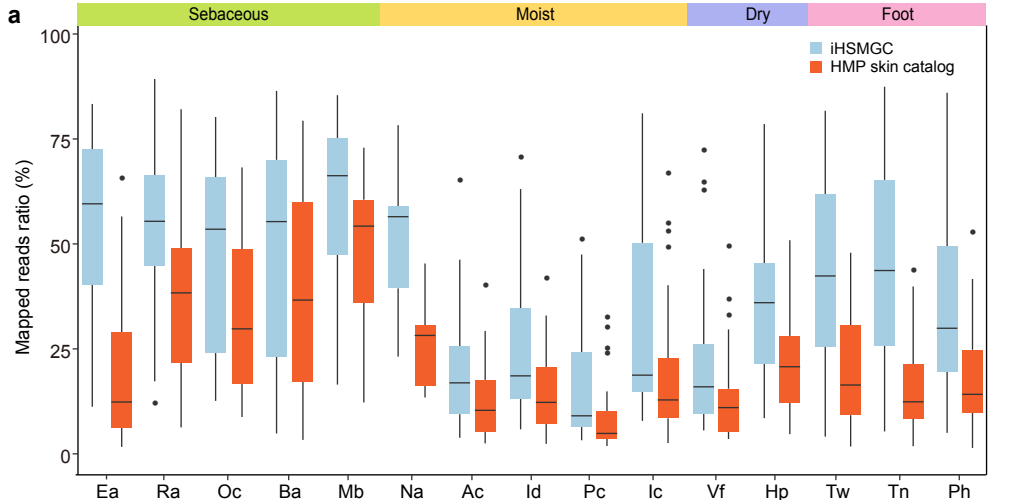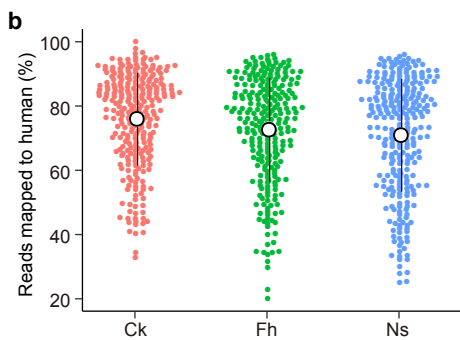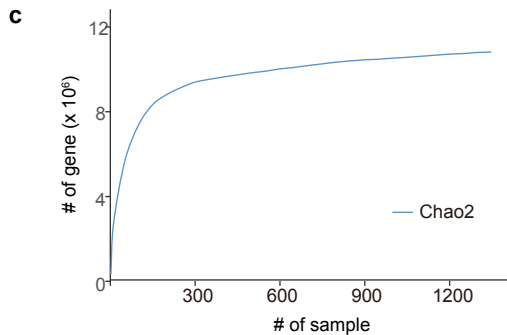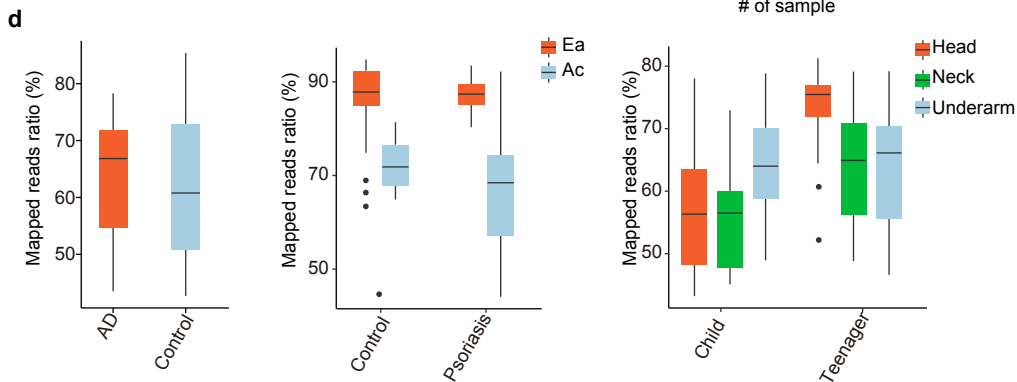

Supplement: Supplementary file 4 — Additional file 3: Figure S2. Host information, coverage and completeness of the iHSMGC. a, Box plot comparing the reads mapping rate of the HMP dataset between HMP skin catalog and the iHSMGC. b, The Bee swarm plot showing the percentage of sequenced reads mapping to human hg19 of each sample. Different anatomical sites are indicated by different colors. c, Rarefaction curve based on gene profiles of 1,361 samples using the Chao2 estimator. d, Box plots demonstrating the read mapping rate from the dataset of Singapore Chinese (NCBI No. PRJNA277905), Italians (NCBI No. PRJNA281366) and another Singapore Chinese (NCBI No. PRJEB26427) by using the iHSMGC. AD-atopic dermatitis. Ea-External auditory canal, Ra-Retroarticular crease, Oc-Occiput, Ba-Back, Mb-Manubrium, Na-Nare, Ac-Antecubital fossa, Id-Interdigital web, Pc-Popliteal fossa, Ic-Inguinal crease, Vf-Volar forearm, Hp-Hypothenar palm, Tw-Toe webspace, Tn-Toenail, Ph-Plantar heel. [file 40168_2020_995_MOESM4_ESM.pdf]

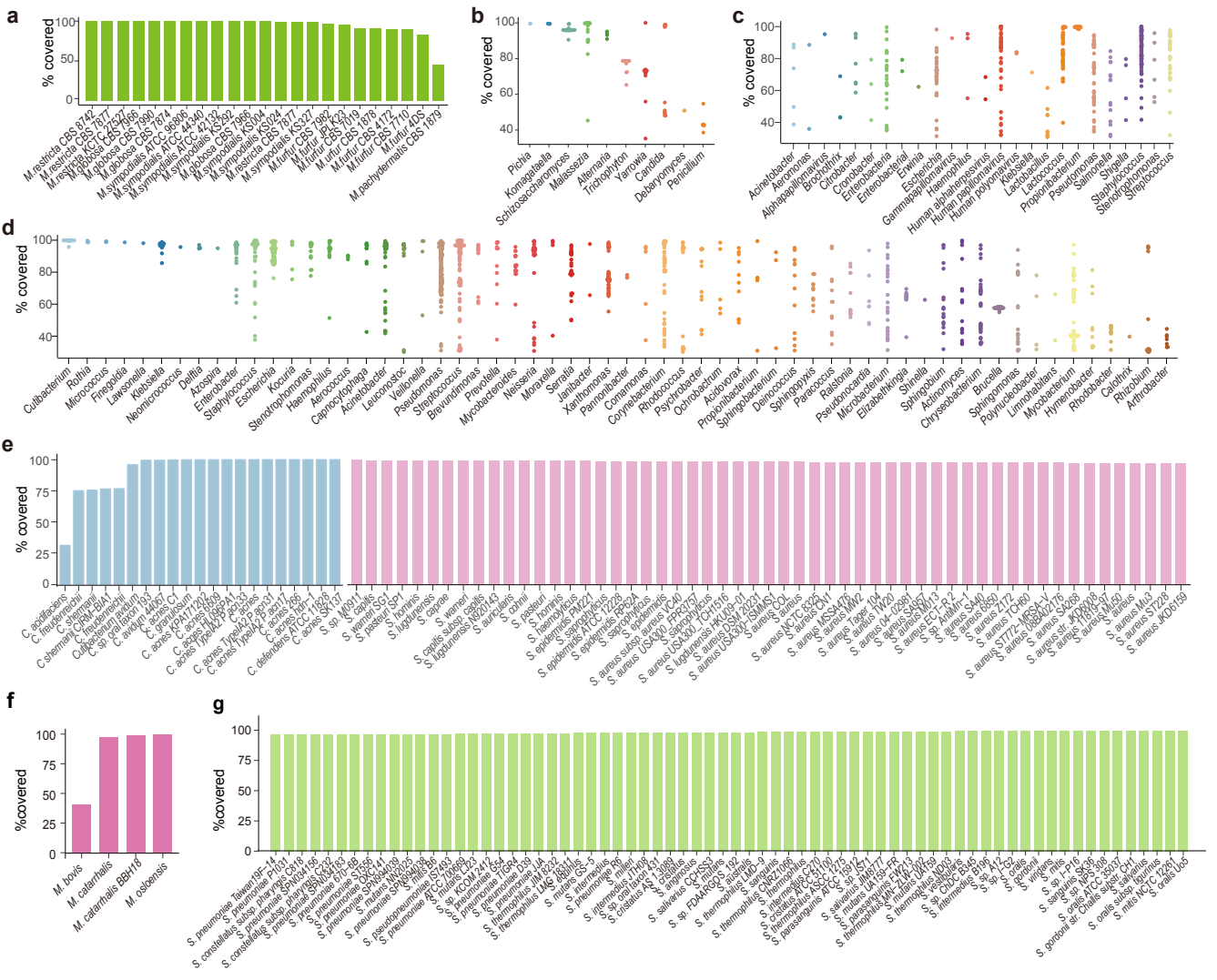

Supplement: Supplementary file 5 — Additional file 4: Figure S3. Evaluation of iHSMGC integrity. Genome coverage of (a) Malassezia sp., (b) top 10 genera of fungi, (c) top 25 genera of viruses, (d) top 60 genera of prokaryotes, each dot in (b-d) represents a species in the genera. Genome coverage of (e) Cutibacterium sp. and Staphylococcus sp., (f) Moraxella sp. and (g) Streptococcus sp. [file 40168_2020_995_MOESM5_ESM.pdf]

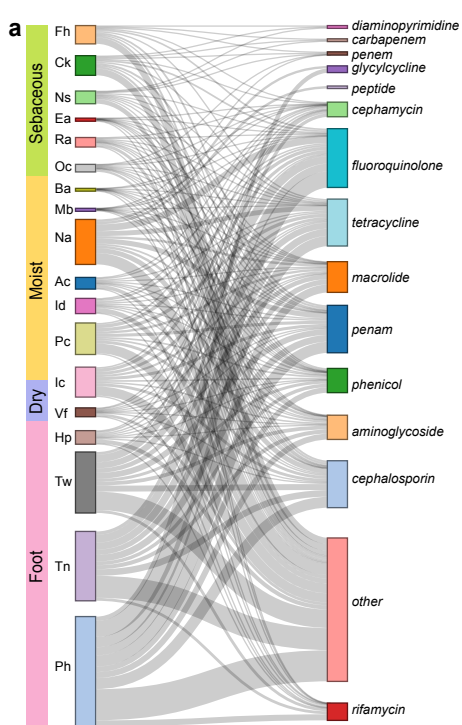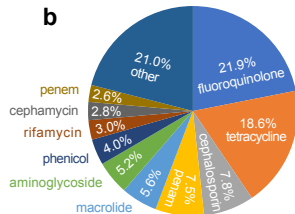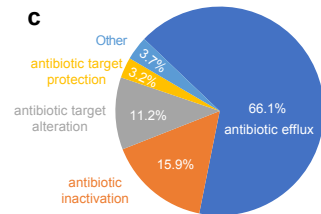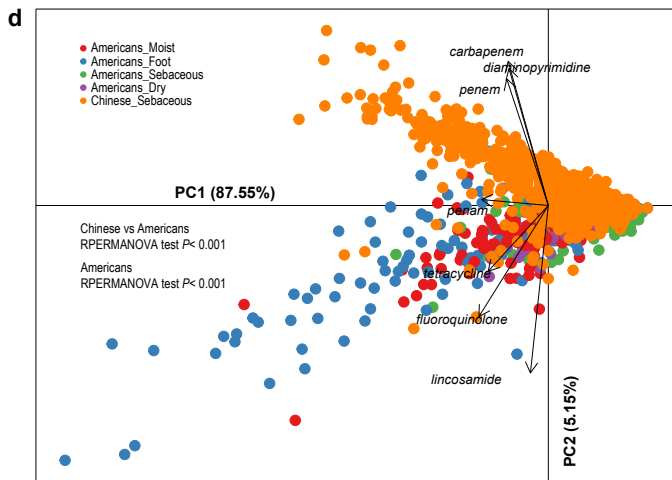

Supplement: Supplementary file 6 — Additional file 5: Figure S4. Drug-resistant spectrum based on ARGs in different skin sites. a, Sankey diagram depicting the distribution of the top 15 types of antibiotics ranked by the corresponding ARG abundance. The height of the rectangles indicates the ARGs relative abundance against the corresponding drug resistance potential within the site. Each site and drug resistance potential is indicated in distinct colors. Fh-Forehead, Ck-Cheek, Ns-Nose, Ea-External auditory canal, Ra-Retroarticular crease, Oc-Occiput, Ba-Back, Mb-Manubrium, Na-Nare, Ac-Antecubital fossa, Id-Interdigital web, Pc-Popliteal fossa, Ic-Inguinal crease, Vf-Volar forearm, Hp-Hypothenar palm, Tw-Toe webspace, Tn-Toenail, Ph-Plantar heel. b-c, The pie chart showing the proportion of drug resistance (b) and resistance mechanisms (c). d, Principal component analysis indicating separation of drug-resistant spectrum within the different anatomical sites. [file 40168_2020_995_MOESM6_ESM.pdf]

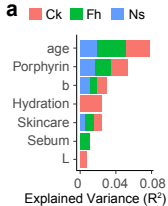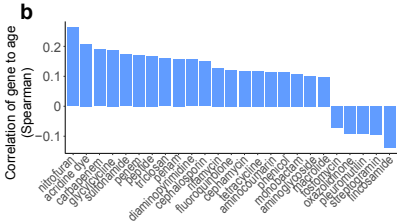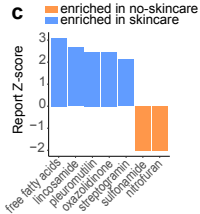

Supplement: Supplementary file 7 — Additional file 6: Figure S5. Factors correlated with drug resistance potential in Chinese. a, Bar chart comparing the explained variance (R2) of factors impacting the relative abundance of ARGs using the Adonis test. The L-value represents skin color from dark to white, the b-value is skin color from blue to yellow. b, Bar chart depicting the types of antibiotics corrected with age by the Spearman correlation. c, Bar chart showing the correlation with skincare habit (p < 0.05). [file 40168_2020_995_MOESM7_ESM.pdf]

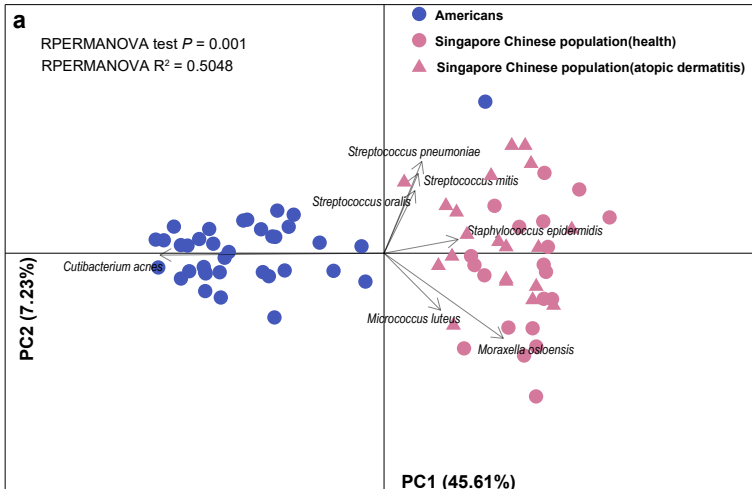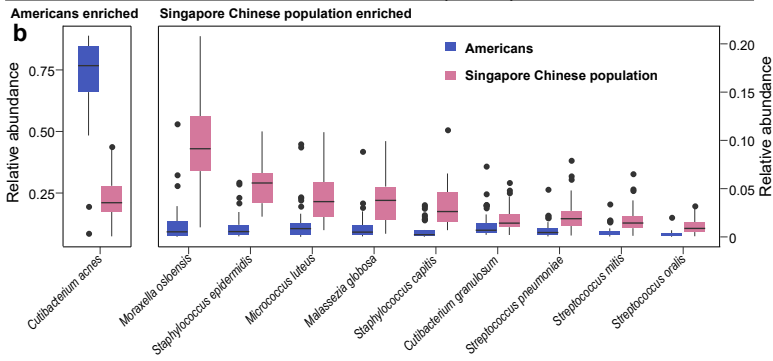

Supplement: Supplementary file 8 — Additional file 7: Figure S6. Differences in skin microbiota between Singapore Chinese and North Americans. a, Principal component analysis presenting the separation of skin microbiota of Singaporean Chinese (NCBI No. PRJNA277905) versus North Americans (HMP SRA bio-project 46333). The microbes, which were the main contributors to the separation, are indicated by arrows. b, The boxplot showing the prominent species that differ significantly in abundance between Singaporean Chinese and North Americans. [file 40168_2020_995_MOESM8_ESM.pdf]

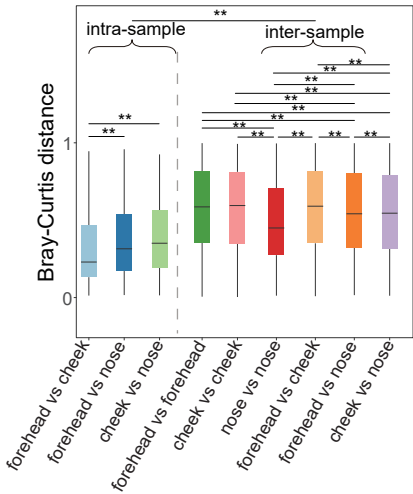

Supplement: Supplementary file 9 — Additional file 8: Figure S7. Intraindividual differences are smaller than interindividual differences. Boxplots of Bray-Curtis distance depicts the similarity between anatomical sites in the face of the same individuals (intraindividual comparisons) or between the same/different sites of different individuals (interindividual comparisons). The left side of the dotted line shows the intraindividual differences, the right side the interindividual differences. The significance levels in the Wilcoxon rank-sum test are: +, p < 0.05; *, p < 0.01; **, p < 0.001. [file 40168_2020_995_MOESM9_ESM.pdf]

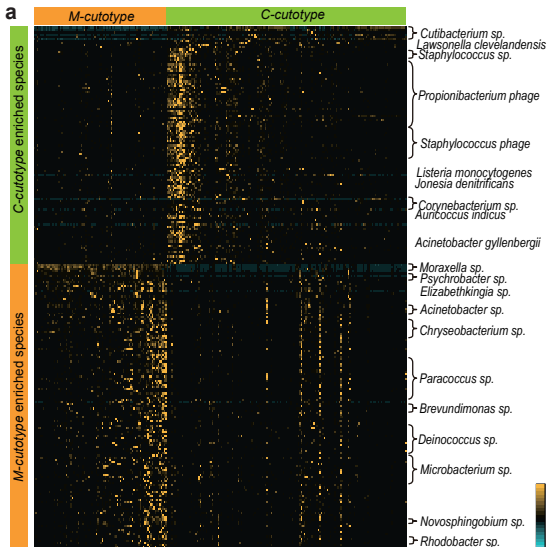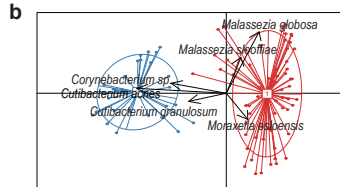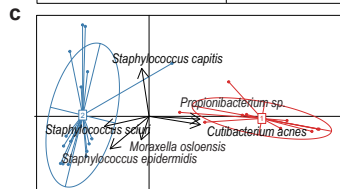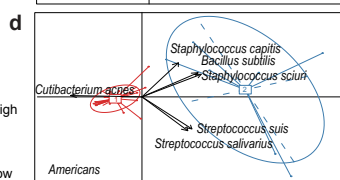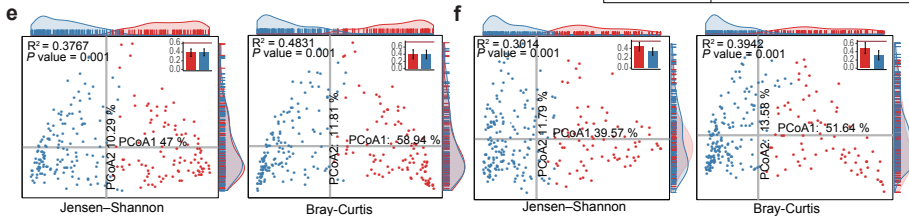

Supplement: Supplementary file 10 — Additional file 9: Figure S8. Microbial composition of the cutotypes and further validation. a, Heat map depicting the species differentially abundant within the two cutotypes (Wilcoxon rank-sum test, p < 0.01). Each lattice represents the relative abundance of the microbe in a sample, yellow indicates high abundance and blue indicates lower abundance. b-d, PCoA using Jensen-Shannon distance presenting the clustering of (b) samples from the Singaporean dataset (NCBI No. PRJEB26427), (c) Samples from the Italian (NCBI No. PRJNA281366) and (d) Samples from the HMP (SRA bio-project 46333). e-f, PCoA using Jensen-Shannon distance and Bray-Cutis dissimilarity presenting the clustering of samples from the cheek (e) and the back of the nose (f) of Han Chinese. Box plots in the top right show the mean distance within the corresponding groups in red or in blue. The red horizontal line indicates the average between-clusters distance. The PERMANOVA test was used to determine the significance between two clusters and is shown in the top left. [file 40168_2020_995_MOESM10_ESM.pdf]

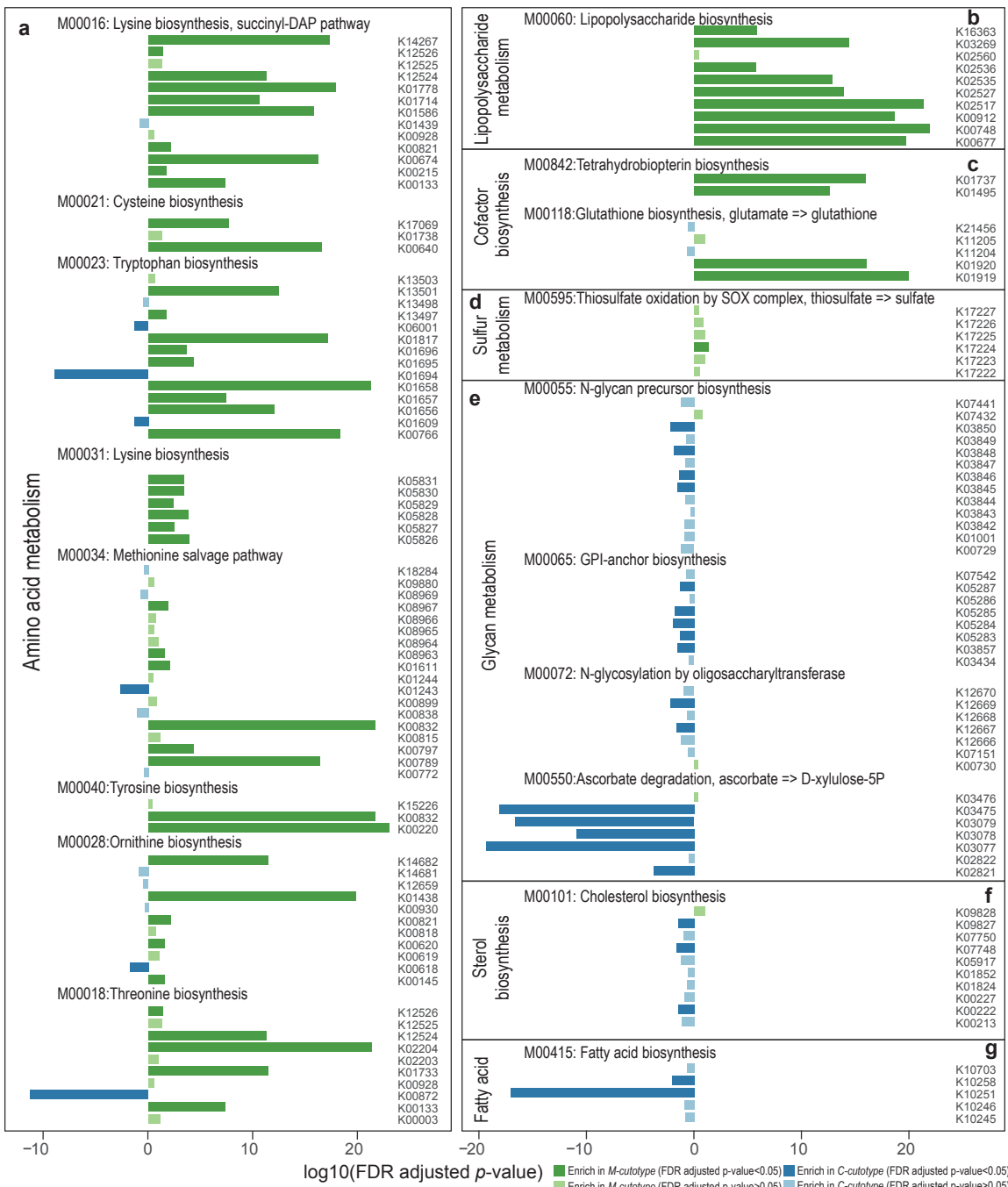

Supplement: Supplementary file 11 — Additional file 10: Figure S9. Microbial functional differences between the two cutotypes. Using log10 (FDR adjusted p-value) bar-plot comparing the abundance of module (amino acid metabolism, lipopolysaccharide metabolism, cofactor biosynthesis, sulfur metabolism, glycan metabolism, sterol biosynthesis, and fatty acid metabolism) KOs of the two cutotypes present in the forehead areas. Green color indicates KO enrichment in the M-cutotype and blue means the enrichment in the C-cutotype. The color shade indicates the level of significance, i.e. dark green or dark blue equal the FDR adjusted p-value < 0.05, which is the threshold for a significant difference. [file 40168_2020_995_MOESM11_ESM.pdf]

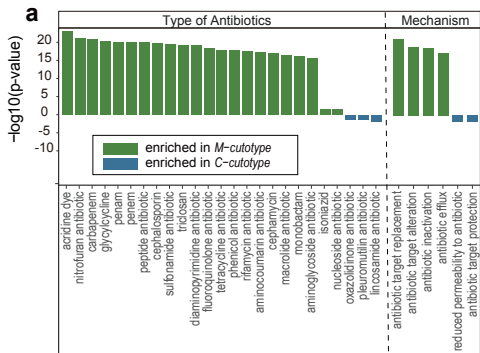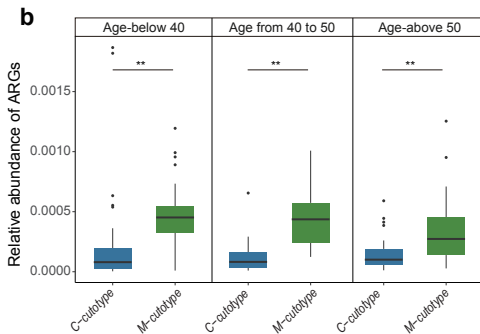

Supplement: Supplementary file 12 — Additional file 11: Figure S10. Characteristics of different skin microbial cutotypes a, Alterations in skin microbial ARGs antibiotics and ARGs mechanism. b, The boxplot showing the differences in the abundance of ARGs between different age groups. Blue, C-cutotype-enriched; green, M-cutotype-enriched. The significance levels in the Wilcoxon test are denoted as: **, p < 0.01. [file 40168_2020_995_MOESM12_ESM.pdf]
