# Supplementary material for: Characterization of the human skin resistome and identification of two microbiota cutotypes
Source: Microbiome. 2021 Feb 17;9:47. doi: 10.1186/s40168-020-00995-7 (PMC7890624; doi:10.1186/s40168-020-00995-7)
Supplement: Supplementary file 13 — Additional file 12: Figure S11. Octylphenol polyethoxylates transformation and beta-carotene biosynthesis between the two skin cutotypes. a, Reaction step for the conversion of octylphenol polyethoxylates to alkylphenol ethoxylates. The histogram compares the abundance of the genes encode for the two enzymes within the two cutotypes (* p< 0.05, ** p < 0.01; Wilcoxon test). b, Reaction steps for the biosynthesis of beta-carotene in microorganisms. The green box represents the enrichment of the KOs in the M-cutotype, the white box represents no significant difference. [file 40168_2020_995_MOESM13_ESM.pdf]

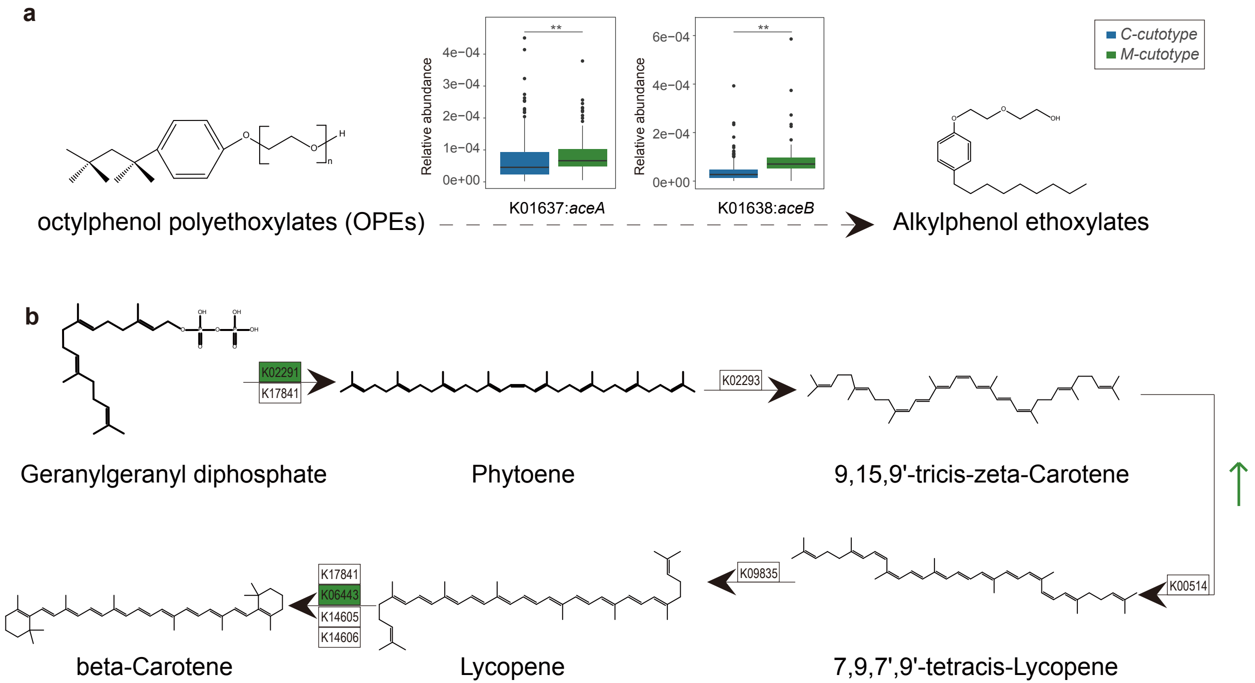

a
Relative abundance
4e−04
3e−04
2e−04
1e−04
0e+00
**
K01637:aceA
6e−04
4e−04
2e−04
0e+00
**
K01638:aceB
C-cutotype
M-cutotype
octylphenol polyethoxylates (OPEs)
Alkylphenol ethoxylates
b
K02291
K17841
K02293
K00514
K09835
K17841
K06443
K14605
K14606
Geranylgeranyl diphosphate
Phytoene
9,15,9'-tricis-zeta-Carotene
7,9,7',9'-tetracis-Lycopene
Lycopene
beta-Carotene
